# Ethanol Stimulates Locomotion via a G_αs_-Signaling Pathway in IL2 Neurons in *Caenorhabditis elegans*

**DOI:** 10.1534/genetics.117.300119

**Published:** 2017-09-25

**Authors:** James R. Johnson, Mark R. Edwards, Huw Davies, Daniel Newman, Whitney Holden, Rosalind E. Jenkins, Robert D. Burgoyne, Robert J. Lucas, Jeff W. Barclay

**Affiliations:** * The Physiological Laboratory, Institute of Translational Medicine, University of Liverpool, L69 3BX, UK; † Faculty of Biological and Medical Sciences, University of Manchester, M13 9PT, UK; ‡ Department of Molecular and Clinical Pharmacology, Institute of Translational Medicine, University of Liverpool, L69 3BX, UK

**Keywords:** alcohol, HSF1, optogenetics, protein kinase A, UNC-18

## Abstract

Alcohol is a potent pharmacological agent when consumed acutely at sufficient quantities and repeated overuse can lead to addiction and deleterious effects on health. Alcohol is thought to modulate neuronal function through low-affinity interactions with proteins, in particular with membrane channels and receptors. Paradoxically, alcohol acts as both a stimulant and a sedative. The exact molecular mechanisms for the acute effects of ethanol on neurons, as either a stimulant or a sedative, however remain unclear. We investigated the role that the heat shock transcription factor HSF-1 played in determining a stimulatory phenotype of *Caenorhabditis elegans* in response to physiologically relevant concentrations of ethanol (17 mM; 0.1% v/v). Using genetic techniques, we demonstrate that either RNA interference of *hsf-1* or use of an *hsf-1*(*sy441*) mutant lacked the enhancement of locomotion in response to acute ethanol exposure evident in wild-type animals. We identify that the requirement for HSF-1 in this phenotype was IL2 neuron-specific and required the downstream expression of the α-crystallin ortholog HSP-16.48. Using a combination of pharmacology, optogenetics, and phenotypic analyses we determine that ethanol activates a G_αs_-cAMP-protein kinase A signaling pathway in IL2 neurons to stimulate nematode locomotion. We further implicate the phosphorylation of a specific serine residue (Ser322) on the synaptic protein UNC-18 as an end point for the G_αs_-dependent signaling pathway. These findings establish and characterize a distinct neurosensory cell signaling pathway that determines the stimulatory action of ethanol and identifies HSP-16.48 and HSF-1 as novel regulators of this pathway.

## Abstract (precis)

Alcohol abuse is among the top causes of preventable death, generating considerable financial, health, and societal burdens. Paradoxically, alcohol...

Alcohol is one of the most prevalent addictive substances and its overuse produces a severe burden on society (WHO 2011). Acute effects of alcohol include motor incoordination, sedation, and anesthesia. Alcohol also acts as a stimulant, activating neurotransmitter release within the brain’s reward circuitry, increasing heart rate, and inducing aggression and risk-taking (Hendler *et al.* 2013). The cellular mechanisms underlying either the anesthetic or stimulatory effects of ethanol are only now being elucidated. Ethanol was initially hypothesized to act through indirect perturbations of the lipid environment thereby affecting broadly all membrane protein function (Harris *et al.* 2008). Recent work instead implicates more direct effects on specific protein targets, particularly membrane proteins such as ion channels and receptors (Howard *et al.* 2014; Trudell *et al.* 2014). Ethanol has a very simple molecular structure and is thought to exert its intoxicating effects through interactions with these target proteins with very low affinity (∼0.1 M) to alter the natural dynamics of protein function (Howard *et al.* 2011; Olsen *et al.* 2014). A complete understanding of both the acute and chronic effects of alcohol within the nervous system is an important unresolved question in physiology.

Invertebrates are excellent genetic models for the identification of cellular/molecular mechanisms governing the sedative properties of ethanol and other anesthetics (Barclay *et al.* 2010; Devineni and Heberlein 2013; Bettinger and Davies 2014). In *Drosophila*, studies of acute ethanol intoxication have implicated a large number of proteins and signaling pathways (Kaun *et al.* 2012). In *Caenorhabditis elegans*, proteins such as Munc18 and Rab3 (Kapfhamer *et al.* 2008; Graham *et al.* 2009; Johnson *et al.* 2013), the BK channel *slo-1* (Davies *et al.* 2003), the leak channel regulator *Lightweight* (Speca *et al.* 2010), chloride intracellular channels (Bhandari *et al.* 2012), and the α-crystallin ortholog HSP-16.48 (Johnson *et al.* 2016) all affect sensitivity to high levels of ethanol (400 mM). The use of very high external ethanol in *C. elegans* behavioral analysis is thought to be a consequence of poor penetration through the nematode cuticle (Alaimo *et al.* 2012). Indeed, internal concentrations in response to 400 mM external ethanol are estimated between 20 and 60 mM (Alaimo *et al.* 2012; Johnson *et al.* 2016), although not all studies are in agreement (Mitchell *et al.* 2007). In addition to sedation, at lower concentrations ethanol acts as a stimulant (Phillips and Shen 1996; Wolf *et al.* 2002; Balino *et al.* 2016). In *C. elegans*, ethanol-induced stimulation occurs at external concentrations (17 mM, 0.1%) whose absolute values would be physiologically consistent with blood alcohol limits for impaired driving (House of Commons Transport Committee 2010). Acute exposure of nematodes to 17 mM ethanol causes a small, but characteristic increase in locomotion rate (Graham *et al.* 2009; Johnson *et al.* 2013). Virtually nothing is known about the cellular and molecular basis underlying *C. elegans* phenotypes at this ethanol concentration.

Heat shock activates a transcriptional response to toxic insults whereby protective cellular chaperone (heat shock protein) expression is increased under the control of the heat shock transcription factor (HSF1) (Anckar and Sistonen 2011). The HSF1-HSP pathway is also ubiquitously involved in stress-independent cellular functions such as polypeptide folding, protein–protein interactions (Kampinga and Craig 2010), and proteostasis (Morimoto 2011), as well as contributing to diseases like cancer (Mendillo *et al.* 2012) and neurodegeneration (Kondo *et al.* 2013). In *C. elegans*, RNA interference (RNAi) or loss-of-function mutations of *hsf-1* increase stress sensitivity, but also accelerate ageing (Hsu *et al.* 2003; Prahlad *et al.* 2008; Baird *et al.* 2014). We have recently shown that *hsf-1* loss-of-function increases sensitivity to 400 mM ethanol (Johnson *et al.* 2016). This hypersensitivity was partially the result of the downstream basal expression of HSP-16.48, an ortholog of the human small heat shock protein α-crystallin, in a process unrelated to an HSF-1-dependent heat shock stress response. Here, we identify that the stimulatory ethanol phenotype also required HSF-1, but specifically in six IL2 chemosensory neurons and could be completely rescued by transgenic expression of HSP-16.48 in IL2 neurons of the *hsf-1* mutant. Using a combination of pharmacology, genetics, and optogenetics, we determined further that this ethanol-dependent stimulation of motility acts via a G_αs_-cAMP-protein kinase A (PKA) signaling pathway within the IL2 sensory neurons and identifies the exocytotic protein UNC-18 as a downstream effector for PKA. Although individual components of the G_αs_ pathway have been linked previously to the neuronal effects of ethanol, this study uniquely characterizes the entire signaling pathway in ethanol-dependent stimulation.

## Materials and Methods

### Nematode culturing, strains, and genetics

*C. elegans* were cultured under standard conditions at 20° on Nematode Growth Media (NGM) agar plates with OP50 *Escherichia coli* as a food source as previously described (Brenner 1974; Graham *et al.* 2009; Edwards *et al.* 2012). These experiments used the following strains: Bristol N2 (wild-type), PS3551 *hsf-1(sy441)*, KG524 *gsa-1(ce94)*, KG421 *gsa-1(ce81)*, MT363 *goa-1(n363)*, NM1380 *egl-30(js126)*, KG1180 *lite-1(ce314)*, and NL2099 *rrf-3(pk1426)*. To investigate effects of single-copy transgenic rescue of *hsf-1(sy441)* mutants, we analyzed the OG532 (*hsf-1(sy441);drSi13[hsf-1p*::*hsf-1*::*GFP*::*unc-54 3′UTR + Cbr-unc-119(+)]*) and OG580 (*hsf-1(sy441);drSi28[hsf-1p*::*hsf-1(R145A)*::*GFP*::*unc-54 3′UTR + Cbr-unc-119(+)]*) strains (Morton and Lamitina 2013), which are single-copy mos1-mediated Single Copy Insertion (MosSCI)-produced rescues of the *hsf-1(sy441)* mutant. Basal locomotion rates for each strain can be found in Supplemental Material, Table S1 in File S1. Transgenic animals were derived by germline injection as previously described (Mello *et al.* 1991; Graham *et al.* 2009; Edwards *et al.* 2012). All injections were performed with 10 ng/µl (indicated construct) and 30 ng/µl (indicated co-injection marker), and made up to 100 ng/µl in total with empty filler DNA (either pUC19 or pBluescript). For each transgenic strain, three independent lines were isolated and tested phenotypically. The results presented were consistent for all individual lines; however, individual line results can be found in Table S2 in File S1. Transgenic lines used in this study include the following: N2;*ulvEx[P_hsf-1_*::*hsf-1]*, N2;*ulvEx[P_rab-3_*::*hsb-1]*, N2;*ulvEx[P_klp-6_*::*hsb-1]*, N2;*ulvEx[P_rab-3_*::*hsp-16.48]*; N2;*ulvEx[P_rab-3_*::*hsp-16.48* Δ*38-44]*, N2;*ulvEx[P_klp-6_*::*hsp-16.48]*, N2;*ulvEx[P_unc-18_*::*unc-18 S322A]*, N2;*ulvEx[P_klp-6_*::*unc-18 S322A]*, N2;*ulvEx[P_klp-6_*::*kin-1*::*P_klp-6_]*, *hsf-1(sy441);ulvEx[P_hsf-1_*::*hsf-1]*, *hsf-1(sy441);ulvEx[P_rab-3_*::*hsf-1]*, *hsf-1(sy441);ulvEx[P_myo-3_*::*hsf-1]*, *hsf-1(sy441);ulvEx[P_glr-1_*::*hsf-1]*, *hsf-1(sy441);ulvEx[P_unc-17_*::*hsf-1]*, *hsf-1(sy441);ulvEx[P_osm-6_*::*hsf-1]*, *hsf-1(sy441);ulvEx[P_gcy-8_*::*hsf-1]*, *hsf-1(sy441);ulvEx[P_klp-6_*::*hsf-1]*, *hsf-1(sy441);ulvEx[P_rab-3_*::*hsp-16.48]*, *hsf-1(sy441);ulvEx[P_rab-3_*::*hsp-16.48* Δ*38-44]*, and *hsf-1(sy441);ulvEx[P_klp-6_*::*hsp-16.48]*. Transgenic constructs in Bristol N2 were coexpressed with either a *P_sur-5_*::*GFP* or a *P_rab-3_*::*GFP* marker. To indicate appropriate cellular expression for additional cell–tissue-specific promoters, each of these transgenics used a GFP coexpression marker under the control of the same promoter. IL2 neurons were visualized with the promoter::GFP reporter *hsf-1(sy441);ulvEx[P_klp-6_*::*GFP*; *P_myo-2_*::*mCherry]*. To examine cell-specific RNAi, Bristol N2 worms were injected with the plasmid *P_klp-6_*::*kin-1*::*P_klp-6_*, which drove cell-specific expression of *kin-1* (catalytic subunit of *C. elegans* protein kinase A) in both the forward and reverse direction (Esposito *et al.* 2007).

### RNAi experiments

RNAi experiments were performed using the *rrf-3(pk1426)* strain. RNAi was induced by feeding (Kamath and Ahringer 2003) using the ORFeome-based RNAi library (Rual *et al.* 2004) as described previously (Johnson *et al.* 2016). HT115 RNAi bacteria were cultured in LB media with 100 μg/ml ampicillin and spotted onto 60 mm diameter NGM plates supplemented with 1 mM isopropyl β-1-thiogalactopyranoside and 25 μg/ml carbenicillin. NGM plates were dried for 4 days before spotting. Five L3–L4 worms were added to each RNAi plate and cultured at 20°. Phenotypic analysis was performed on first-generation progeny fed with the indicated RNAi bacterial clones. For the negative control for the RNAi screen, worms were fed with an empty feeding vector.

### Cloning

*C. elegans* genes of interest were amplified from either Bristol N2 genomic DNA (*hsp-16.48*) or cDNA (*hsf-1*), cloned into pDONR201 and recombined into DEST vectors to create tissue-specific expression vectors, as previously described (Johnson *et al.* 2016). For *hsb-1*, the gene was amplified from Bristol N2 genomic DNA using the following primers:

*hsb-1* attB forward: 5ʹ-GGGGACAAGTTTGTACAAAAAAGCAGGCTTCATGTCCGATGAGAAGTCTACC-3ʹ.
*hsb-1* AttB reverse:
5ʹ-GGGGACCACTTTGTACAAGAAAGCTGGGTCTTATTGAGCGCTTGGCGGATGTTC-3ʹ.

Mutagenesis of the *hsp-16.48* gene was performed using a Q5 Site-Directed Mutagenesis Kit (New England Biolabs, Beverly, MA) as per the manufacturer’s instructions. The *unc-18* S322A expression vector was created using the Gene Tailor mutagenesis kit (Invitrogen, Carlsbad, MA) from a vector carrying the *unc-18* cDNA under the control of the *unc-18* genomic flanking regions (gift from H. Kitayama, Kyoto University, Japan) (Gengyo-Ando *et al.* 1996). To create the *P_klp-6_*::*unc-18* construct, the existing *unc-18* promoter was excised by *Eco*RI and *Bam*HI restriction digest and replaced with a *klp-6* promoter using the NEBuilder DNA assembly (New England Biolabs). For glutathione-S-transferase (GST) fusion protein production, *unc-18* was subcloned into pGEX-6p-1 as previously described (Edwards *et al.* 2012).

All *C. elegans* promoter fragments were amplified from Bristol N2 genomic DNA and cloned into pPD117.01 (kind gift of A. Fire, Stanford University) in place of P*_mec-7_*. These vectors were converted into Gateway DEST vectors using a conversion cassette (Life Technologies). *P_rab-3_* (kind gift of M. Nonet, Washington University in St. Louis), *P_myo-3_* (kind gift of A. Fire, Stanford University), and *P_hsf-1_* are previously described (Edwards *et al.* 2012; Johnson *et al.* 2016). The following primers were used for additional promoter cloning:

*P_unc-17_* forward: 5ʹ-AGTCGGCGCGCCATCCGTTCCCATCCGCTTCATC-3ʹ.
*P_unc-17_* reverse: 5ʹ-AGGAGGATCCGGTTACTATTTTGAACAAGAGATGCGG-3ʹ.
*P_glr-1_* forward: 5ʹ-AGTCGGCGCGCCCTGTAGCCGGTATGCACTGATAAC-3ʹ.
*P_glr-1_* reverse: 5ʹ-AGTCGGATCCTGTGAATGTGTCAGATTGGGTG-3ʹ.
*P_osm-6_* forward: 5ʹ-ATGTGGCGCGCCCAGTGGAATCACCATTGGGTATCCAG-3ʹ.
*P_osm-6_* reverse: 5ʹ-GGGTGGATCCGAAGGTAATAGCTTGAAAGAGATATAAGCCC-3ʹ.
*P_gcy-8_* forward: 5ʹ-AGTCGGCGCGCCAACTACCTTCCTCCGCGTCC-3ʹ.
*P_gcy-8_* reverse: 5ʹ-AGTCGGATCCTTTGATGTGGAAAAGGTAGAATCG-3ʹ.
*P_klp-6_* forward: 5ʹ-CCCCGGCGCGCCAACGTCCCAGACAATTTCAAC-3ʹ.
*P_klp-6_* reverse: 5ʹ-CTACGGATCCGGAGTCACCCTTTCCCCTTATTCTG-3ʹ.

The *P_klp-6_*::*kin-1*::*P_klp-6_* construct was made as follows. A 750-bp fragment of *kin-1* was amplified from the Vidal library clone (Rual *et al.* 2004) and subcloned into the *P_klp-6_:*:GFP expression vector, downstream of *P_klp-6_* in place of GFP, using the NEBuilder cloning kit (New England Biolabs). The *P_klp-6_*::*kin-1* PCR fragment was then amplified from this construct and again subcloned into the same *Pklp-6* construct as before, but in reverse orientation. Correct construction was confirmed by PCR and sequencing. Primers used were as follows:

*kin-1* RNAi forward: 5ʹ-CTATCGATTCGCGGCCATCACAAGTTCGAATCGGA-3ʹ.
*kin-1* RNAi reverse (step 1): 5ʹ-CTTGTGGGCTTTTGTATAGTTCGTCCGGATAAC-3ʹ.
*kin-1* RNAi reverse (step 2): 5ʹ-CTATCGATTCGCGGCCATCACAAGTTGGATAACTAC-3ʹ.

### Protein phosphorylation and mass spectrometry

For *in vitro* biochemistry, recombinant proteins (GST, GST-UNC-18) were produced as described previously (Edwards *et al.* 2012). For phosphorylation experiments, 2 μg of substrate protein was incubated with 2 units of PKA catalytic subunit (Sigma [Sigma Chemical], St. Louis, MO), 100 μM ATP, and 2 μCi [γ-^32^P]ATP (GE Healthcare) in a 50 μl final reaction volume of 2-(N-morpholino)ethanesulfonic acid (MES) buffer (50 mM MES, 10 mM MgCl_2_, 1 mM DTT, and 0.5 mM EDTA, pH 6.9). Reactions were incubated at 30° for 3 hr before termination. To determine phosphorylation, 20 μl of the kinase reaction was separated by SDS-PAGE, stained with Coomassie Blue dye, destained overnight in a destainer [35% ethanol, 2% glycerol (v/v)], air-dried in Hoeffer Easy Breeze plastic frames (Thermo Fisher Scientific), exposed to a phosphor screen for 2–4 hr, and scanned by a PhosphorImager 425 (Molecular Dynamics). For *in vitro* phosphorylation site determination, phosphorylated samples were separated on NuPAGE 4–12% Bis-Tris precast gels (Invitrogen) and stained with Coomassie Blue dye before excision of protein bands. Gel plugs were destained in 50% acetonitrile (v/v)/50 mM ammonium bicarbonate and dried before incubation in trypsin (5 ng/μl in 50 mM ammonium bicarbonate) for 16 hr at 37°. Peptides were then extracted by sonication of gel plugs in 60% (v/v) acetonitrile/1% (v/v) trifluoroacetic acid. Extracts were thoroughly dried and ZipTipped (Millipore, Bedford, MA) before electrospray ionization mass spectrometry (MS). Residual peptides were resuspended in 50% (v/v) acetonitrile/0.05% trifluoroacetic acid, and 5 μl of suspension was delivered into a QStar Pulsar I hybrid quadrupole time-of-flight MS (AB Sciex) by automated in-line liquid chromatography [integrated LCPackings System, 5 mm C18 nano-precolumn, and 75 μm × 15 cm C18 PepMap column (Dionex)]. A gradient from 5 to 48% acetonitrile/0.05% trifluoroacetic acid (v/v) in 60 min was applied at a 300 nl/min flow rate and survey scans of 1 sec were acquired for m/z 400–2000. The most intense ions were selected for tandem MS with 2 sec accumulation times and a dynamic exclusion of 30 sec. Identification and analysis was performed using MASCOT software (Matrix Science).

### Optogenetics

JellyOp (Bailes *et al.* 2012) and hRh1 (Bailes and Lucas 2013) coding domain sequences were PCR amplified from pcDNA3.1 and pcDNA3.5 expression vectors, respectively, and subcloned downstream of *P_klp-6_* in pPD117.01 (detailed above) via Gibson Cloning techniques (New England Biolabs). The optogenetic vectors were then transformed by germline injection into *C. elegans* as described above. The vectors were expressed in the *lite-1(ce314)* background to prevent any potential photophobic responses (Husson *et al.* 2013). All optogenetic worm strains were then grown in the dark at 20° on standard NGM plates with OP50 bacteria as a food source. One day prior to assays, late L4 worms were picked onto standard 60 mm NGM plates with 50 μl OP50 supplemented with 100 μM 9-*cis*-Retinal, an active chromophore for opsin. All plates and worms were handled either in the dark or under a dim red light (> 630 nm). Thrashing assays were performed under the dim red light in Dent’s solution (140 mM NaCl, 6 mM KCl, 1 mM CaCl_2_, 1 mM MgCl_2_, and 5 mM HEPES, pH 7.4, with bovine serum albumin at 0.1 mg/ml), in the presence or absence of 17 mM ethanol, using a Leica MZ10F-stereomicroscope (Leica, UK) equipped with a pE-300 light-emitting diode (LED) fluorescence light source (CoolLED, UK). To activate opsin, worms were illuminated with a single flash of a 100% power green LED through the ET GFP filter set for 5 sec. For hRh1, opsin activation occurred as soon as they were placed in the Dent’s solution and thrashing was quantified after 10 min acclimation (as below). For JellyOp, worms were illuminated following the 10 min acclimation in Dent’s solution just prior to thrashing quantification, as the JellyOp activation is more transient (Bailes *et al.* 2012). Assays were repeated on three separate days to confirm replicability.

### Behavioral assays

Phenotypic analysis was performed in a temperature-controlled room on young adult hermaphrodites from sparsely-populated plates grown at 20°. Unless otherwise indicated, experiments were conducted at an ambient temperature of 20°. Here, locomotion rate was measured as thrashing (Gjorgjieva *et al.* 2014) in 200 μl Dent’s solution as previously described (Graham *et al.* 2009; Johnson *et al.* 2013). One thrash was defined as a complete movement from maximum to minimum amplitude and back again. For acute ethanol experiments, ethanol was diluted to 17 mM in Dent’s solution, and locomotion was quantified following 10 min exposure and normalized as a percentage of the mean thrashing rate of untreated worms measured each day (at least 10 control worms per strain). For forskolin (Sigma) experiments, forskolin was diluted in dimethyl sulfoxide (DMSO) and added to Dent’s solution in the indicated concentration. For direct comparison in these experiments, control (untreated) worms were exposed to Dent’s with an equal concentration of DMSO. H-89 (Sigma) was diluted in water and added to the Dent’s/DMSO solution in the indicated concentration. For chemotaxis assays (Kashyap *et al.* 2014), experimental assay plates (100 mm Petri dishes) contained 2% (w/v) agar, 5 mM KH_2_PO_4_, 1 mM CaCl_2_, and 1 mM MgSO_4_. Plates were poured 3 days prior to use and allowed to dry. Worms were washed in M9 buffer and placed on the center of the plate. Next, 1 μl of the attractant (100% ethanol) and a negative control (distilled water) were pipetted on opposite sides of the plate. The number of worms on either side of the plate was calculated following 90 min. The chemotaxis index (C.I.) was calculated as C.I. = (# worms at attractant − # worms at control)/total number of worms. For worm avoidance assays, a 0.9-cm ring of a substance was made by a stamping procedure on unseeded NGM plates. Immediately after stamping, 30 worms were placed within the center of the ring, completed within 2 min. Following 10 min exposure, the number of worms that had crossed the ring was counted. In preliminary experiments, 0.9 cm was found to be sufficient to permit most worms to cross a neutral substance (distilled water) within 10 min. All data are expressed as mean ± SE. As indicated, significance was tested by Student’s *t*-test, Mann–Whitney *U*-test, or ANOVA with Tukey *post hoc* comparisons where appropriate.

### Data availability

Strains are available upon request. The authors state that all data necessary for confirming the conclusions presented in the article are represented fully within the article.

## Results

**Figure 1:**
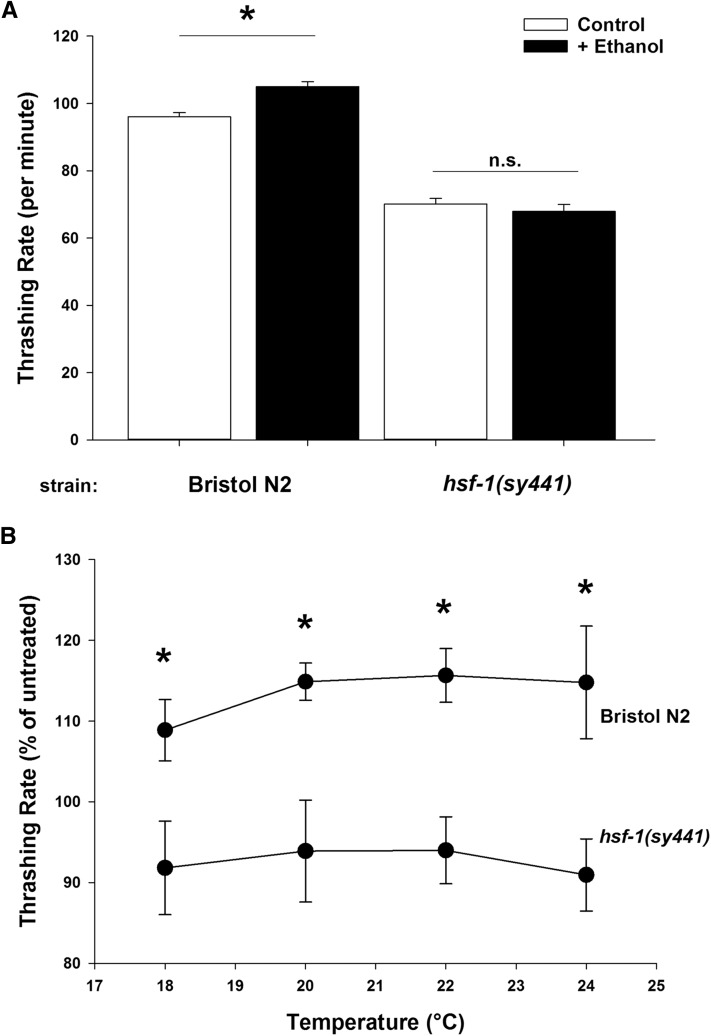
Low concentrations of ethanol stimulate *C. elegans* locomotion in an *hsf-1*-dependent fashion. (A) In comparison to untreated controls, exposure to 17 mM ethanol (+ Ethanol) significantly increased the locomotion of Bristol N2 wild-type worms. The *hsf-1(sy441)* loss-of-function mutant demonstrated a reduced untreated locomotion rate that was unaffected by ethanol. Locomotion rate was quantified by thrashing after 10 min immersion in Dent’s solution. * *P* < 0.001 (one-way ANOVA with Tukey *post hoc* comparisons); n.s. = not significant. *N* = 100 for each condition. (B) The absence of ethanol stimulation in *hsf-1(sy441*) worms is independent of temperature. Locomotion rate was quantified at the indicated temperature and expressed here as a percentage of mean thrashing rate of untreated worms. At all temperatures, ethanol stimulated Bristol N2 locomotion, but had no effect on *hsf-1(sy441)* loss-of-function worms. * *P* < 0.001 (two-way ANOVA with Tukey *post hoc* comparisons); *N* = 20 for each condition.

HSF-1, the heat shock transcription factor, is involved in a plethora of cellular functions (Anckar and Sistonen 2011; Morimoto 2011; Vihervaara and Sistonen 2014). In *C. elegans*, the *hsf-1(sy441)* allele is a viable loss-of-function point mutation in the *hsf-1* gene that acts as an inhibitor of HSF-1 transcriptional activity (Hajdu-Cronin *et al.* 2004), increasing temperature sensitivity and decreasing lifespan (Baird *et al.* 2014). Nematodes respond to high external ethanol concentration (400 mM) by a dose-dependent decrease in coordinated locomotion (Davies *et al.* 2003; Graham *et al.* 2009) and we have recently characterized a novel role for HSF-1 in determining this phenotype (Johnson *et al.* 2016). However, worms additionally respond phenotypically to low levels of external ethanol (17 mM) with a reproducible enhancement in locomotor activity (Graham *et al.* 2009; Johnson *et al.* 2013). In Bristol N2 wild-type worms, this stimulation is a 5–10% increase in basal locomotion rate, as quantified by thrashing (Figure 1A). We tested whether the *hsf-1(sy441)* mutation would also hypersensitize worms to the effects of low concentrations of ethanol. Surprisingly, we found that the stimulatory effect of 17 mM external ethanol was completely absent in worms containing the *hsf-1(sy441)* mutation (Figure 1A). The *hsf-1(sy441)* mutant is well established to have a temperature-sensitive phenotype and we verified that the low-dose ethanol phenotype was not affected by the ambient temperature (Figure 1B); however, all other described experiments were conducted at 20° standard temperature.

**Figure 2:**
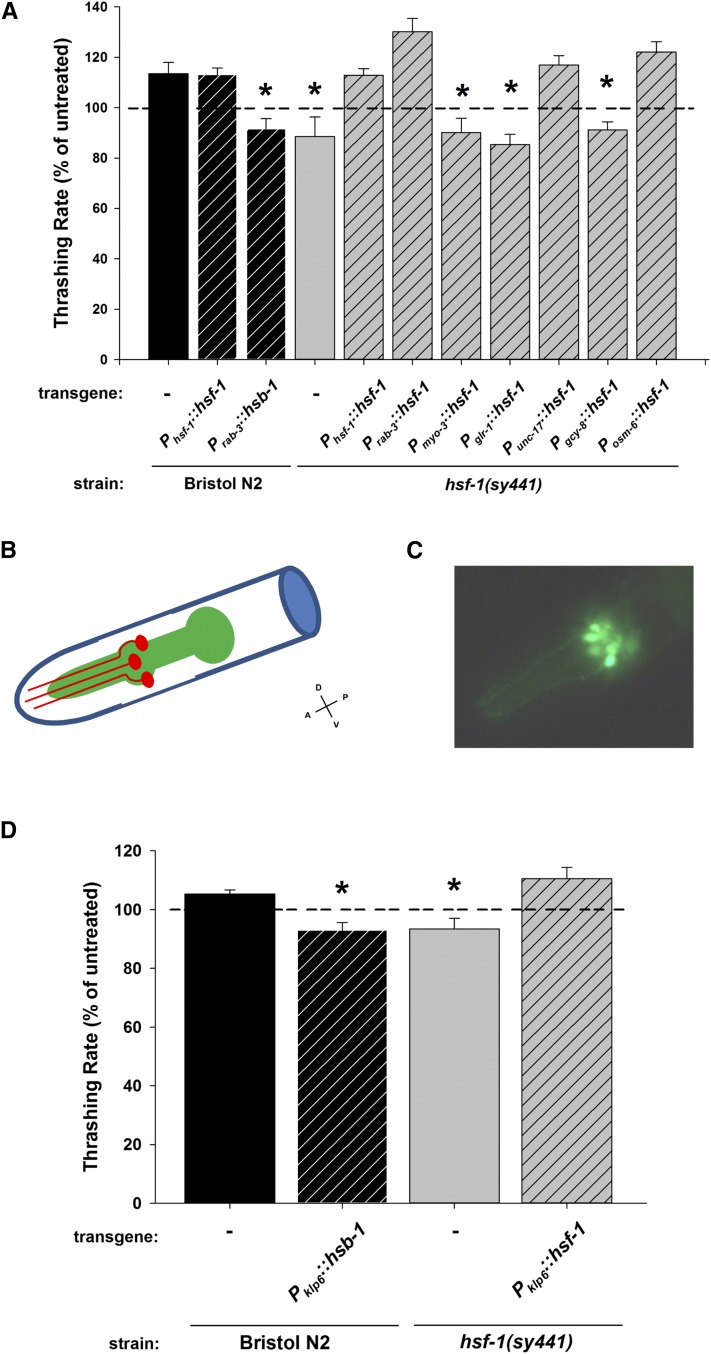
The *hsf-1* dependence of the low ethanol stimulation of locomotion requires *hsf-1* expression in IL2 neurons. (A) In *hsf-1(sy441)* worms, the low ethanol stimulation of locomotion was restored by transgenic rescue of wild-type *hsf-1* under the control of its endogenous (*P_hsf-1_*), pan-neuronal (*P_rab-3_*), cholinergic neuron (*P_unc-17_*), or ciliated sensory neuron promoter (*P_osm_*_-6_). Tissue-specific expression in muscle (*P_myo-3_*), interneurons (*P_glr-1_*), or the AFD thermosensory neurons (*P_gcy-8_*) was unable to rescue the *hsf-1(sy441)* mutant. In Bristol N2 worms, overexpression of *hsf-1* under its endogenous promoter (*P_hsf-1_*) had no additional effect on ethanol stimulation of locomotion, whereas neuronal overexpression of the *hsf-1* inhibitor, *hsb-1*, blocked the ethanol effect. (B) Cartoon schematic of the *C. elegans* head region indicating the location of the IL2 neurons (one neuron of each of the three pairs are depicted) and their projections (in red) adjacent to the pharynx (in green). The approximate anterior–posterior (A–P) and ventral–dorsal (V–D) axes are indicated and apply to part C as well. (C) Expression of green fluorescent protein (GFP) under the control of the *klp*-6 promoter, showing expression in the IL2 neurons of *hsf-1(sy441)* mutants. Expression in Bristol N2 worms was anatomically similar. (D) In comparison to *hsf-1(sy441)* worms, low ethanol stimulation of locomotion was restored by transgenic expression of wild-type *hsf-1* using an IL2-specific promoter (*P_klp-6_*). In Bristol N2 worms, IL2 neuron overexpression of the *hsf-1* inhibitor, *hsb-1*, blocked the ethanol effect. In both (A) and (D), data are expressed normalized to untreated controls. For each experiment, exposure to ethanol enhanced the locomotion rate of Bristol N2 worms (Mann–Whitney *U*-test; *P* < 0.05). * indicates significant difference in comparison to treated Bristol N2. Comparisons were made by one-way ANOVA with Tukey *post hoc* comparisons (*P* < 0.001; *N* = 30 for each condition). Bristol N2 worms are depicted in black, *hsf-1(sy441)* in gray. Hatching indicates transgenic expression (transgene and promoter indicated below graph).

HSF-1 is a ubiquitously expressed transcription factor that is active in all cell types; however, the role of *hsf-1(sy441)* in the hypersensitivity to 400 mM external ethanol is dependent upon pan-neuronal *hsf-1* expression (Johnson *et al.* 2016). Therefore, we next investigated next whether the ablation of the low (17 mM) ethanol stimulation of locomotion phenotype observed in the *hsf-1(sy441)* mutant was also pan-neuronal in nature by tissue-specific transgenic rescue. To allow for a direct comparison of the stimulatory effects of ethanol independent of basal locomotor rates, the data are presented as thrashing rate normalized to untreated worms of the same strain as done in previous studies (Davies *et al.* 2003; Graham *et al.* 2009; Johnson *et al.* 2016). However, basal rates for all strains can be compared in Table S1 in File S1. While overexpression of *hsf-1* in Bristol N2 controls had no additional effect, transgenic rescue of *hsf-1(sy441)* under its own promoter (*P_hsf-1_*::*hsf-1*) or specifically under the control of a pan-neuronal promoter (*P_rab-3_*::*hsf-1*) completely rescued the stimulatory ethanol phenotype (Figure 2A). However, transgenic rescue of *hsf-1(sy441)* using a body wall muscle promoter (*P_myo-3_*::*hsf-1*) was insufficient to rescue. As a complementary approach to investigate *hsf-1* function in the ethanol phenotype, we cloned and overexpressed *hsb-1* in Bristol N2. *hsb-1* is a negative regulator for *hsf-1* transcriptional function and its overexpression in *C. elegans* results in similar phenotypes to loss-of-function of *hsf-1* (Satyal *et al.* 1998). Overexpression of *hsb-1* pan-neuronally in Bristol N2 worms (*P_rab-3_*::*hsb-1*) also resulted in a loss of ethanol stimulation of locomotion similar to that seen in the *hsf-1*(*sy441)* mutant (Figure 2A). From these transgenic rescue experiments, we conclude that the loss of the ethanol stimulation phenotype is a consequence of a loss in neuronal *hsf-1* function.

As the *rab-3* promoter drives expression throughout the nervous system, we next determined whether the stimulatory ethanol phenotype could be localized more precisely in the nervous system. Intriguingly, the 17-mM ethanol stimulatory phenotype of *hsf-1(sy441)* worms could be reestablished by transgenic expression of wild-type *hsf-1* specifically in cholinergic neurons (*P_unc-17_*::*hsf-1*) or in ciliated sensory neurons (*P_osm-6_*::*hsf-1*), but not in interneurons (*P_glr-1_*::*hsf-1*) or the AFD thermosensory neurons (*P_gcy-8_*::*hsf-1*) (Figure 2A). The localization of the ethanol-induced stimulation of locomotion to either cholinergic neurons or ciliated sensory neurons was particularly serendipitous, as these two promoters are predicted to overlap in only one nematode cell type, the inner labial sensilla neurons, IL2 (Figure 2B). There are three pairs of IL2 neurons located in the head of the animal, all with ciliated endings (White *et al.* 1986; Burket *et al.* 2006). IL2 neurons are cholinergic (Zhang *et al.* 2014) and are predicted to be chemosensory as their dendrites are exposed to the external environment. Very little has been published ascribing function to the IL2 neurons; however, they do regulate nictation, a behavior whereupon a nematode waves its head in three dimensions (Lee *et al.* 2012). We tested whether the absence of the stimulatory ethanol phenotype of *hsf-1(sy441)* could be reversed by transgenic expression of wild-type *hsf-1* specifically in the IL2 neurons. For this, we cloned the promoter region of the *klp-6* gene, which in hermaphrodites is expressed specifically in the IL2 neurons (Peden and Barr 2005). Localization was verified by examination of a promoter::GFP transgenic worm, confirming that the IL2 neuronal structures appeared anatomically intact even in the *hsf-1*(*sy441)* mutant (Figure 2C), although an in-depth ultrastructural analysis of the cilia in this mutant remains to be fully investigated. We then determined that expression of wild-type *hsf-1* in the IL2 neurons (*P_klp-6_*::*hsf-1*) alone could restore the stimulatory ethanol phenotype of the *hsf-1(sy441)* mutant to a level indistinguishable from wild-type worms (Figure 2D). The complementary approach also confirmed that IL2-specific overexpression of *hsb-1* (*P_klp-6_*::*hsb-1*), the negative regulator for *hsf-1*, was able to block the ethanol-induced stimulation in Bristol N2 (Figure 2D).

*hsf-1(sy441)* worms have a slightly lower basal locomotor rate (Figure 1A); however, previous transgenic rescue experiments indicate that the basal locomotion rate is uncorrelated with the high ethanol phenotype (Johnson *et al.* 2016). Our data here also support a lack of correlation between basal locomotor rate and the low-ethanol phenotype (Table S1 in File S1). For example, IL2 neuron overexpression of *hsb-1* in Bristol N2 does not reduce basal locomotion rate at all, but does block the low-ethanol phenotype. Alternatively, overexpression of wild-type *hsf-1* in IL2 neurons in the *hsf-1(sy441)* mutant background does not rescue the locomotor defect of the mutant, but did restore the low-ethanol phenotype. Notwithstanding this lack of correlation, we were interested in determining whether the reduced locomotor rate in the *hsf-1(sy441)* mutant was a consequence of the point mutation. The *hsf-1(sy441)* strain was originally isolated in a genetic screen of suppressors of heat shock-induced expression, where it was both backcrossed and outcrossed (Hajdu-Cronin *et al.* 2004). We have demonstrated here (Table S1 in File S1) and elsewhere (Johnson *et al.* 2016) that transgenic rescue can alter many *hsf-1*-dependent phenotypes, without altering the basal locomotor rates, consistently indicating that the defect in locomotion may be independent of the *sy441* mutation or possibly incomplete rescue by the transgene. To address this question more directly we analyzed the OG532 strain, which is a single-copy MosSCI-integrated rescue of *hsf-1(sy441)* (Morton and Lamitina 2013). The OG532 strain demonstrated a significant, yet incomplete, rescue of the locomotion defect in comparison to Bristol N2 (Table S1 in File S1). The incomplete rescue of the locomotion defect was reminiscent of a reported incomplete rescue of the *hsf-1(sy441)* temperature-sensitivity (Morton and Lamitina 2013). Reassuringly, the single-copy rescue did restore the ethanol stimulatory phenotype (Figure S1) to a level similar to that seen with transgenic overexpression. In contrast, the OG580 strain, a single-copy rescue of *hsf-1(sy441)* with a DNA-binding defective mutant *hsf-1* (Morton and Lamitina 2013), impaired locomotion further (Table S1 in File S1) and did not rescue the ethanol phenotype (Figure S1). These data indicate a potentially complex role for *hsf-1* in basal locomotor rate, which is perhaps unsurprising given the broad cellular functions of the HSF-1 transcription factor and its downstream transcriptional targets. Critically, however, our results show that the basal locomotor rate is unrelated to the ethanol stimulatory effect and is instead a consequence of the *hsf-1(sy441)* mutation.

**Figure 3:**
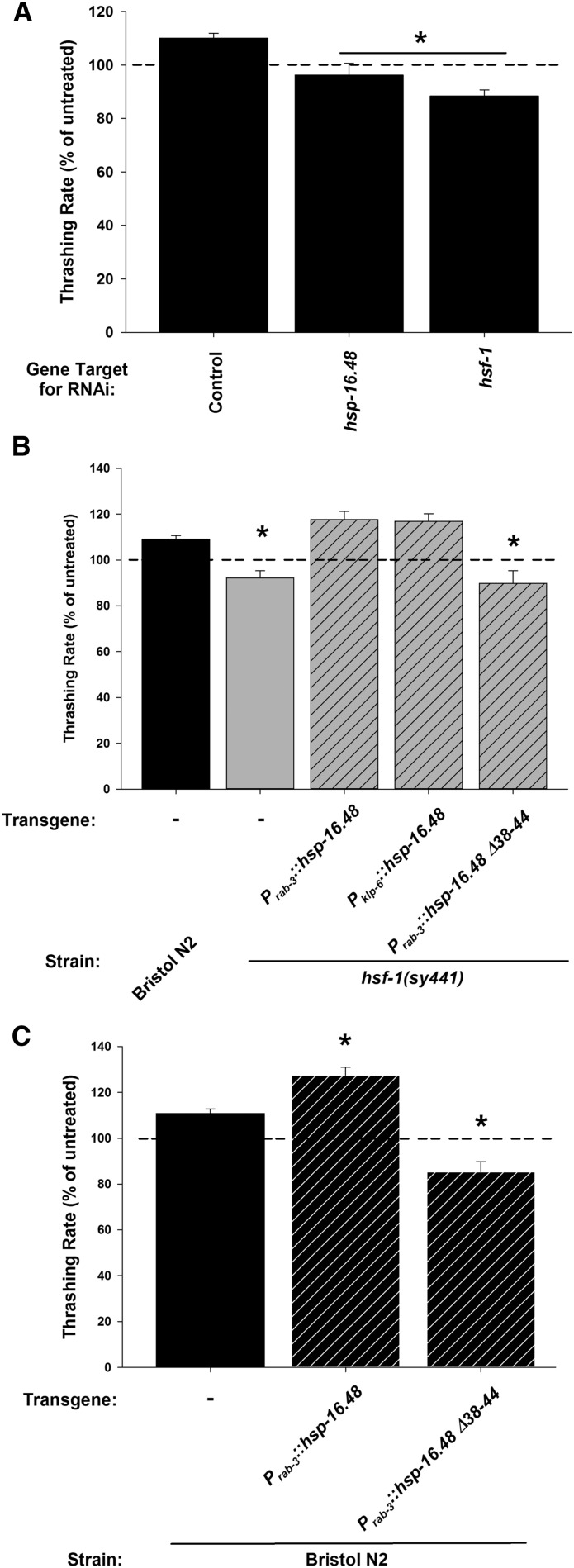
HSP-16.48 acts downstream of HSF-1 in IL2 neurons. (A) RNA interference (RNAi) knockdown of the small heat shock protein *hsp-16.48* statistically phenocopied the ethanol sensitivity of *hsf-1* RNAi knockdown in comparison to control. RNAi was performed by feeding where controls were fed empty vector. n.s. = not significant. (B) Either pan-neuronal (*P_rab-3_*) or IL2-specific (*P_klp-6_*) expression of *hsp-16.48* rescued the *hsf-1(sy441)* mutant phenotype. Expression of an *hsp-16.48* truncation mutant (+Δ38-44) was unable to rescue. (C) Overexpression of *hsp-16.48* in Bristol N2 worms enhanced the ethanol stimulation of locomotion to a significantly greater extent, whereas the *hsp-16.48* truncation mutant (+Δ38-44) blocked the ethanol enhancement of locomotion. For (A–C), data are expressed normalized to untreated controls. For each experiment, exposure to ethanol enhanced the locomotion rate of RNAi control or Bristol N2 worms (Mann–Whitney *U*-test; *P* < 0.05). * indicates significant difference in comparison to treated RNAi control or Bristol N2. Comparisons were made by one-way ANOVA with Tukey *post hoc* comparisons [*P* < 0.001; *N* = 10 (A), 30 (B) and 20 (C) for each condition]. Bristol N2 worms are depicted in black, *hsf-1(sy441)* in gray. Hatching indicates transgenic expression (transgene and promoter indicated below graph).

Our previous work on HSF-1 and its effect on sensitivity to 400 mM external ethanol indicated that the main effector for HSF-1 was the small heat shock protein HSP-16.48 (Johnson *et al.* 2016). This work showed that RNAi of *hsp-16.48* phenocopied RNAi of *hsf-1* and that transgenic expression of *hsp-16.48* partially rescued the high-dose ethanol sensitivity of the *hsf-1(sy441)* mutants. Finally, overexpression of *hsp-16.48* greatly reduced wild-type worm sensitivity to high external ethanol in a manner dependent upon an intact seven amino acid region of the N-terminus (amino acids 38–44) (Johnson *et al.* 2016). We next performed a series of experiments to test for functional similarities with the stimulatory ethanol phenotype. There are unfortunately no available *hsp-16.48* null mutations as the gene has undergone evolutionary genetic duplication, rendering null mutations prohibitively difficult to isolate (Johnson *et al.* 2016). Our results showed that RNAi knockdown of *hsf-1* also resulted in a loss of the 17 mM ethanol phenotype (Figure 3A), to an extent similar to that seen with the *sy441* point mutation. Reminiscent of the sedative ethanol phenotype results (Johnson *et al.* 2016), RNAi of *hsp-16.48* also blocked the stimulatory ethanol phenotype to an equivalent level to *hsf-1* RNAi (Figure 3A). However, distinct from the sedative ethanol phenotype (Johnson *et al.* 2016), transgenic expression of *hsp-16.48* either pan-neuronally (*P_rab-3_*::*hsp-16.48*) or in IL2 neurons specifically (*P_klp-6_*::*hsp-16.48*) was sufficient to restore completely the stimulatory ethanol phenotype in the *hsf-1(sy441)* mutant (Figure 3B). Finally, we determined that overexpression of *hsp-16.48* in Bristol N2 wild-types (*P_rab-3_*::*hsp-16.48*) increased the effect of 17 mM external ethanol, whereas the N-terminal truncation mutant (*P_rab-3_*::*hsp-16.48* Δ*38-44*) appeared to act as neomorphic or as dominant-negative (Figure 3C). However, expression of the truncation mutant in the *hsf-1(sy441)* background did not result in an enhancement of the ethanol-dependent reduction in locomotion in comparison to *hsf-1(sy441)* alone (Figure 3B). Therefore, we hypothesize that the truncation mutant is possibly acting here as a dominant-negative. These experiments implicate HSP-16.48 in the stimulatory ethanol phenotype, acting downstream of HSF-1 in a manner requiring the seven amino acid region of the N-terminus.

As HSF-1/HSP-16.48 was acting in a chemosensory neuron to increase locomotion, we were interested to determine whether this increase in motility was reflective of either positive or negative chemotaxis. Indeed, the performed thrashing assays immersed the animals directly into ethanol where it would be impossible to determine any directionality to movement. We tested for this possibility by a performing a chemotaxis assay on wild-type animals toward or away from ethanol. Ethanol has been previously reported to have mild to negative effects on chemotaxis only at very high concentrations (Bargmann *et al.* 1993; Lee *et al.* 2009). We found that, in our hands, ethanol did not act as either a chemoattractant or repellent whereas butanol, a longer-chain alcohol, did act as a strong attractant (Figure S2A) as has been previously described (Bargmann *et al.* 1993). As an alternative approach, we adapted an osmotic aversion assay (Solomon *et al.* 2004) to investigate whether populations of worms would be averse to crossing a ring of ethanol. We found that if the ethanol concentration of the ring was increased to 100%, we could induce avoidance in comparison to a neutral (distilled water) control; however, 17 mM (0.1%) ethanol had no effect (Figure S2B). In contrast, worms were averse to octanol at concentrations as low as 1%.

**Figure 4:**
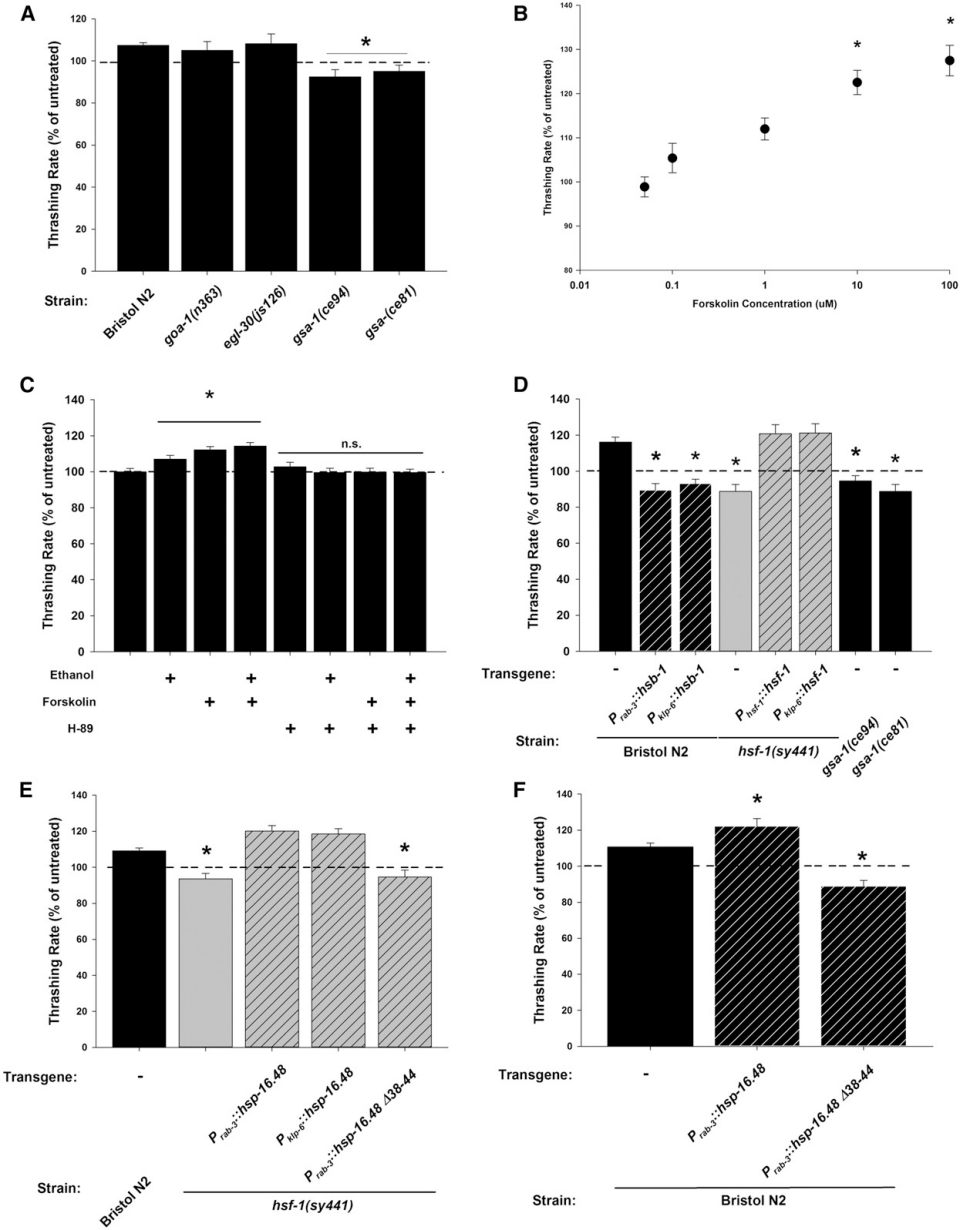
Ethanol enhances locomotion through an IL2 neuron G_αs_-dependent signaling pathway. (A) Stimulation of locomotion by ethanol was blocked in G_αs_ mutants (*gsa-1(ce94)* and *gsa-1(ce81)*), but not in G_ao_ (*goa-1 (n363)*) or G_aq_ (*egl-30(js126)*) mutants. (B) Forskolin-stimulated *C. elegans* Bristol N2 locomotion. Increasing concentrations of forskolin significantly (*) stimulated locomotion at 10 and 100 μM in comparison to untreated worms. (C) Ethanol and forskolin stimulate locomotion of Bristol N2 worms to an equivalent level and this stimulation can be blocked by addition of the PKA inhibitor H-89. * indicates significant increase from untreated. n.s = not significant. (D) Forskolin-dependent stimulation also requires IL2 neuron expression of *hsf-1*. The forskolin effect was absent in *hsf-1(sy441*) mutants but could be rescued by reexpression of wild-type *hsf-1* under its endogenous (*P_hsf-1_*) or an IL2-specific (*P_klp-6_*) promoter. Like ethanol, the forskolin effect was blocked in Bristol N2 worms by pan-neuronal (*P_rab-3_*) or IL2 neuron-specific (*P_klp-6_*) expression of the *hsf-1* inhibitor, *hsb-1*. The forskolin effect was also absent in G_αs_ mutants. (E) Forskolin-dependent stimulation also involved IL2 neuron expression of *hsp-16.48*. Pan-neuronal (*P_rab-3_*) or IL2-specific (*P_klp-6_*) overexpression of *hsp-16.48* rescued the absence of the forskolin phenotype in *hsf-1(sy441*) mutant worms. Expression of the *hsp-16.48* truncation mutant (+Δ38-44) was unable to rescue. (F) Overexpression of *hsp-16.48* in Bristol N2 worms enhanced the forskolin stimulation of locomotion, whereas the truncation mutant (+Δ38-44) acted in a dominant-negative fashion. For each experiment (A–F), data are expressed normalized to untreated controls. Exposure to ethanol or forskolin enhanced the locomotion rate of Bristol N2 worms (Mann–Whitney *U*-test; *P* < 0.05). * indicates significant difference in comparison to treated Bristol N2. Comparisons were made by one-way ANOVA with Tukey *post hoc* comparisons [*P* < 0.001; *N* = 20 (A and B), 25 (C, D, and F) or 30 (E) for each condition]. Bristol N2 worms are depicted in black, *hsf-1(sy441)* in gray. Hatching indicates transgenic expression (transgene and promoter indicated below graph).

We next investigated whether the low concentration of external ethanol was instead activating the IL2 chemosensory neurons to stimulate nematode motility through a G-protein-coupled receptor (GPCR)-dependent signaling mechanism. Physiological effects of ethanol are known to be modulated by various GPCRs, for example the GABA_B_ or G-protein-coupled corticotropin-releasing factor (CRH) 1 receptors (Nie *et al.* 2004; Kelm *et al.* 2011; Agabio and Colombo 2014). However, to screen GPCRs directly would be prohibitively difficult as there are > 1000 GPCRs in the *C. elegans* genome, very few of which have been functionally characterized (Frooninckx *et al.* 2012). Therefore, for simplicity, we instead tested available G-protein mutants. Many loss-of-function nematode G-protein mutants are lethal or have severe locomotor defects, which would interfere with quantification using our assay. Therefore, we screened gain-of-function mutants instead and determined that the stimulatory effect of 17 mM external ethanol remained intact for both G_αo_ (*goa-1*) and G_αq_ (*egl-30*) mutants, but was absent specifically for mutants of G_αs_, *gsa-1(ce81)* and *gsa-1(ce94)* (Figure 4A).

G_αs_ is a G-protein that, upon activation, stimulates adenylyl cyclase to synthesize the cAMP signaling molecule from ATP (Dorsam and Gutkind 2007). We addressed whether we could replicate the stimulatory effect of 17 mM ethanol by applying the chemical forskolin, which directly activates adenylyl cyclase pharmacologically. A dose-response curve of varying forskolin concentrations demonstrated that the stimulatory ethanol phenotype could indeed be phenocopied by forskolin (Figure 4B). Addition of ethanol and forskolin together did not have additive effects on nematode motility (Figure 4C), suggestive that ethanol and forskolin acted in the same pathway. One downstream cellular effector for cAMP is the cAMP-dependent protein kinase PKA and we tested for its role in the stimulatory ethanol phenotype by the addition of the specific PKA inhibitor H-89. On its own, H-89 had no effect on locomotion; however, it could completely block the stimulatory effects of ethanol and forskolin, either separately or both together (Figure 4C).

Next, as previously shown for the stimulatory effects of ethanol, we verified that stimulation by forskolin was also dependent upon IL2 neuron expression of HSF-1 and HSP-16.48. In comparison to Bristol N2 wild-types, the effect of forskolin was absent in *hsf-1(sy441)* mutants, but was completely restored in both the full (*P_hsf-1_*::*hsf-1*) or IL2 neuron-specific transgenic rescues (*P_klp-6_*::*hsf-1*) (Figure 4D). Complementary to these results, stimulation by forskolin was blocked by the *hsf-1* inhibitor *hsb-1* expressed either pan-neuronally (*P_rab-3_*::*hsb-1*) or in IL2 neurons specifically (*P_klp-6_*::*hsb-1*) in Bristol N2 worms. As seen with ethanol, the effect of forskolin was restored in the OG532 single-copy rescue of *hsf-1(sy441)*, but not in the OG580 DNA-binding defective rescue (Figure S1). The forskolin effect was also blocked in the G_αs_ gain-of-function mutants *gsa-1(ce81)* and *gsa-1(ce94)* (Figure 4D). Furthermore, pan-neuronal (*P_rab-3_*::*hsp-16.48*) or IL2-specific (*P_klp-6_*::*hsp-16.48*) overexpression of *hsp-16.48* alone could completely rescue the forskolin phenotype of the *hsf-1(sy441)* mutation (Figure 4E). Also identical to that seen with ethanol, overexpression of *hsp-16.48* in Bristol N2 controls (*P_rab-3_*::*hsp-16.48*) enhanced the effect of forskolin, whereas the N-terminal truncation mutant (*P_rab-3_*::*hsp-16.48* Δ*38-44*) appeared to act negatively (Figure 4F). The truncation mutant again had no additional negative effects when expressed in the *hsf-1(sy441*) background (Figure 4E). Taken together, these data indicate that both the 17 mM ethanol and forskolin stimulatory phenotypes are likely acting through the same pathway. Therefore, ethanol appears to activate a G_αs_-cAMP-PKA signaling pathway that requires *hsf-1* and *hsp-16.48* expression within the IL2 neurons. Additionally, the requirement of HSF-1/HSP-16.48 is likely to be acting downstream of adenylyl cyclase activation as the effect of forskolin is absent in the *hsf-1(sy441)* mutant.

**Figure 5:**
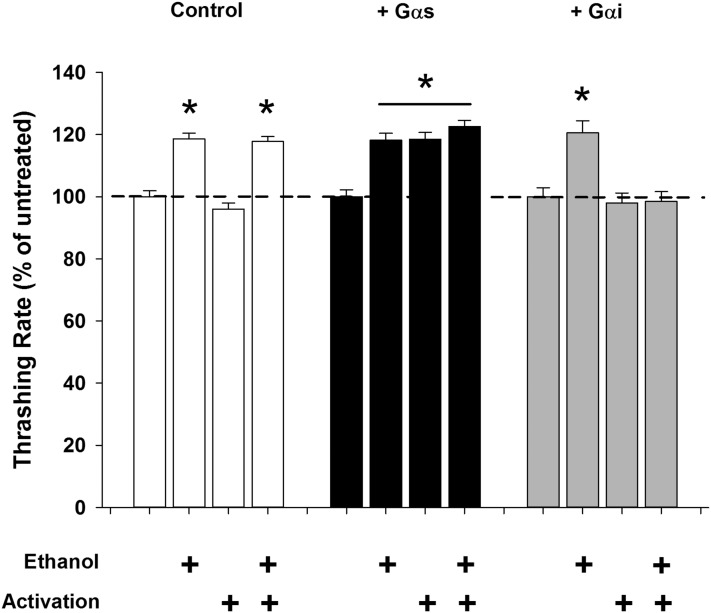
Ethanol enhancement of locomotion requires G_αs_ protein activation in IL2 neurons. Left: in control animals (*lite-1(ce314)*), addition of ethanol enhanced locomotion; however, photostimulation has no stimulatory or depressive effects. Middle: in comparison, IL2-specific optogenetic activation of G_αs_ stimulated *C. elegans* locomotion to an equivalent level to low ethanol exposure (*lite-1(ce314);Ex[P_klp-6_*::*JellyOp]*). Simultaneous ethanol exposure and G_αs_ photoactivation had no additional effect on locomotion. Right: inhibition of G_αs_ by IL2-specific optogenetic activation of G_αi_ blocked ethanol-dependent enhancement of locomotion (*lite-1(ce314);Ex[P_klp-6_*_::_*hRh1]*). Activation of G_αi_ alone had no depressive effect on nematode locomotion. Addition of ethanol without G_αi_ activation exhibited normal locomotor enhancement. All data are expressed normalized to untreated controls. * indicates significant enhancement in comparison to untreated control. Comparisons were made by one-way ANOVA with Tukey *post hoc* comparisons (*P* < 0.001; *N* = 30 for each condition). Control worms (*lite-1(ce314)*) are depicted in white, *lite-1(ce314);Ex[P_klp-6_*::*JellyOp]* in black, and *lite-1(ce314);Ex[P_klp-6_*_::_*hRh1]* in gray.

The data thus far pointed to an activation of G_αs_ in IL2 neurons by 17 mM external ethanol; however, our evidence was obtained by pharmacological activation of the entire nematode. We next wanted to activate G_αs_ in the IL2 neurons directly. To accomplish this, we genetically expressed a photoactivatable G_αs_-linked jellyfish opsin, JellyOp (Bailes *et al.* 2012), in the IL2 neurons specifically and demonstrated that photoactivation could enhance the nematode locomotion rate to a level statistically indistinguishable from ethanol exposure (Figure 5). In contrast, control worms demonstrated no enhancement of locomotion by photostimulation; however, their ethanol effect remained intact. Importantly, photoactivation of the G_αs_-linked JellyOp with the addition of ethanol did not produce a greater summative effect on locomotion rate, supporting the hypothesis that the G_αs_-linked JellyOp and ethanol were acting in the same pathway (Figure 5). Next, we antagonized G_αs_ signaling in IL2 neurons specifically by genetic expression of a photoactivatable G_αi_-linked human rod opsin (hRh1) (Cao *et al.* 2012; Bailes and Lucas 2013), which acts to counter G_αs_ by inhibiting the production of cAMP. On its own, photoactivation of the G_αi_-linked hRh1 again had no effect on nematode locomotion; however, it was able to block completely the stimulatory effect of low concentrations of external ethanol (Figure 5). As an alternative approach to optogenetic activation or inhibition of the G_αs_ signaling pathway, we produced a transgenic worm with IL2 neuron-specific RNAi against the catalytic subunit of *C. elegans* PKA (*P_klp-6_*::*kin-1*::*P_klp-6_*). The created transgenic worms appeared qualitatively normal and had only a small quantitative reduction in basal locomotion rate (Table S1 in File S1). While we cannot quantify definitive cell specificity of the RNAi, if the PKA knockdown had spread greatly to other cells it would be expected to be lethal (Rual *et al.* 2004). Therefore, we are reasonably confident in a degree of cellular restriction of the knockdown. Despite the qualitatively wild-type appearance of the worms, the stimulation of locomotion by either ethanol or forskolin was absent (Figure S3). These data support the interpretation that the effects of ethanol to stimulate *C. elegans* locomotion are regulated in IL2 neurons by a G_αs_ signaling pathway.

**Figure 6:**
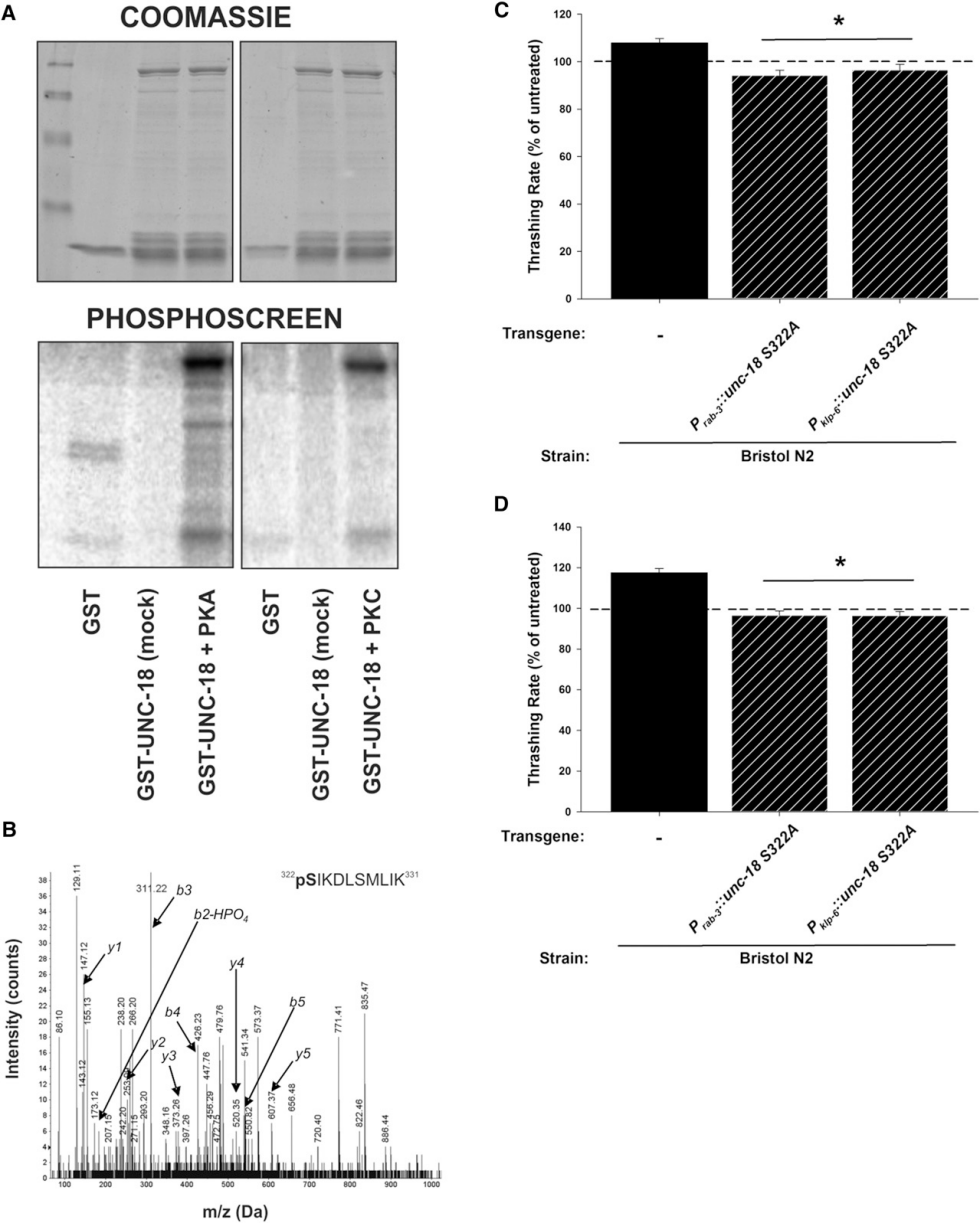
UNC-18 is phosphorylated by protein kinase A, and phosphorylation of UNC-18 Ser322 is required for both ethanol and forskolin-dependent stimulation of locomotion. (A) Recombinant UNC-18 protein (GST-UNC-18) was incubated with ^32^P-labeled ATP in the absence (mock) or presence of either protein kinase C (+PKC) or protein kinase A (+PKA). Proteins were separated by SDS-PAGE and phosphorylation was determined by PhosphorImager (Phosphoscreen). Coomassie staining indicates equal protein loading. (B) Liquid chromatography-tandem mass spectrometry data positively indicating phosphorylation of Ser322 of UNC-18 in PKA-phosphorylated protein samples. (C) Pan-neuronal (*P_unc-18_*) or IL2-specific (*P_klp-6_*) expression of a phospho-null mutation of a PKA-phosphorylation site of *unc-18* (S322A) in Bristol N2 blocked the ethanol-dependent stimulation of locomotion. (D) Pan-neuronal (*P_unc-18_*) or IL2-specific (*P_klp-6_*) expression of a phospho-null mutation of a PKA-phosphorylation site of *unc-18* (S322A) in Bristol N2 blocked the forskolin-dependent stimulation of locomotion. For (C and D), data are expressed normalized to untreated controls. Exposure to ethanol or forskolin enhanced the locomotion rate of Bristol N2 worms (Mann–Whitney *U*-test; *P* < 0.05). * indicates significant difference in comparison to treated Bristol N2. Comparisons were made by one-way ANOVA with Tukey *post hoc* comparisons (*P* < 0.001; *N* = 30 for each condition). Bristol N2 worms are depicted in black. Hatching indicates transgenic expression (transgene and promoter indicated below graph).

Finally we were interested in identifying what was potentially acting downstream of the G_αs_-cAMP-PKA signaling pathway. PKA is a serine/threonine kinase that would be expected to phosphorylate many downstream targets in response to activation. Sec1-Munc18 (SM) proteins are essential components of the exocytotic machinery thought to function primarily through interactions with soluble N-ethylmaleimide-sensitive factor receptor proteins (Rizo and Sudhof 2012). We have previously shown that expression of a single point mutation in the *C. elegans* SM protein UNC-18 (D214N) was also able to block the low-ethanol phenotype (Graham *et al.* 2009). Therefore, we suspected that UNC-18 could be one potential downstream target for PKA phosphorylation. Both mammalian Munc18 and the nematode ortholog UNC-18 are known to be phosphorylated by Protein Kinase C (Barclay *et al.* 2003; Edwards *et al.* 2012); however, PKA phosphorylation has not been previously demonstrated. We expressed recombinant UNC-18 and exposed it to PKA, determining that it could be phosphorylated *in vitro* (Figure 6A). We then determined *in vitro* phosphorylation sites for PKA by MS, positively identifying Ser322 as an *in vitro* PKA target (Figure 6B). We tested the relevance of this putative phosphorylation site *in vivo* by expressing phospho-null versions of UNC-18 (S322A), which act in a dominant fashion to endogenous *unc-18* (Edwards *et al.* 2012). In our assays, expression of this single point mutation of UNC-18 pan-neuronally (*P_unc-18_*::*unc-18 S322A*) or in IL2 neurons specifically (*P_klp-6_*::*unc-18 S322A*) was able to block completely the stimulatory effects on motility of either ethanol or forskolin (Figure 6, C and D).

## Discussion

**Figure 7:**
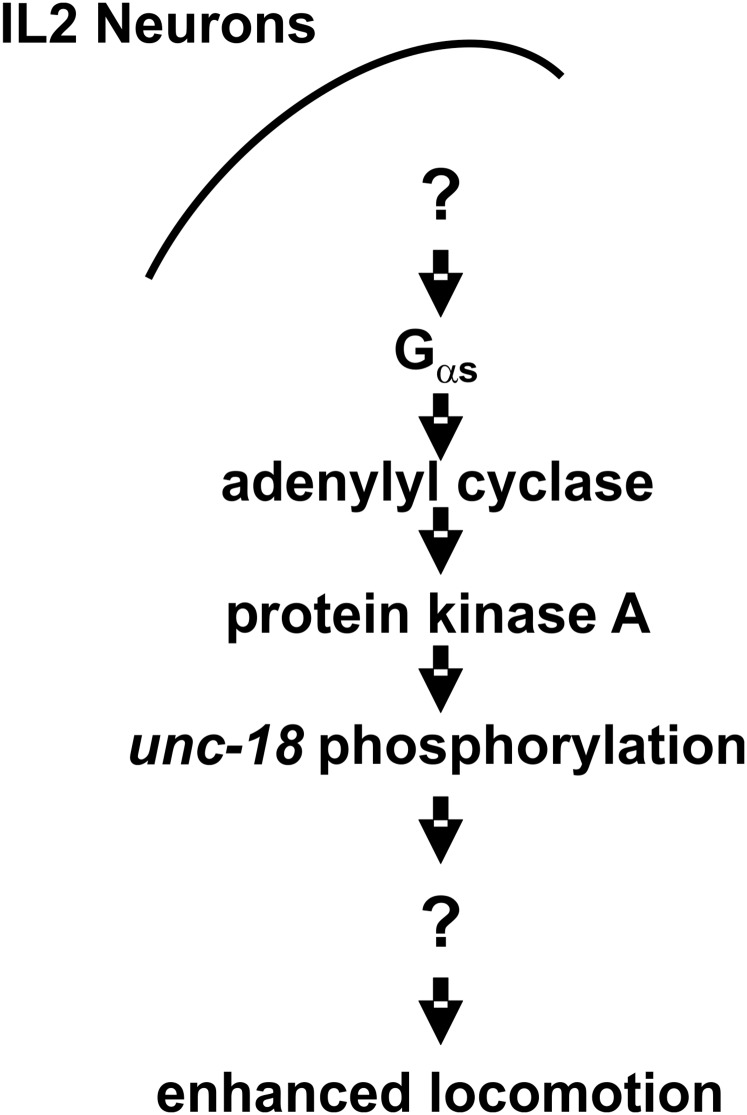
A model for ethanol-dependent enhancement of locomotion in *C. elegans*. The addition of ethanol at a low external concentration (17 mM) activates a G_αs_-dependent signaling pathway in the IL2 chemosensory neurons, likely through an as yet unidentified G-protein coupled receptor. Whether ethanol directly activates or modulates an existing signal remains to be determined. The G_αs_-dependent signaling activates adenylyl cyclase and protein kinase A (PKA). PKA-dependent phosphorylation of the exocytotic protein UNC-18 at Ser322 in some way alters signaling from IL2 neurons, which feeds into the neurons controlling nematode locomotion.

In this manuscript, we demonstrate that a G_αs_-signaling pathway is stimulated by the addition of ethanol at external concentrations (17 mM; 0.1%) whose absolute values are physiologically consistent with blood alcohol limits for impaired driving. Through a combination of genetics, pharmacology, and optogenetic manipulation, we have uniquely delineated the entire cell signaling pathway in response to ethanol specifically in IL2 chemosensory neurons (Figure 7). Our model for ethanol stimulation is that ethanol, in some way, activates G_αs_ to stimulate adenylyl cyclase to produce cAMP. The cAMP in turn activates PKA to phosphorylate various downstream proteins, one possibility of which is the synaptic SM protein UNC-18. The ultimate downstream effect of this cellular signaling pathway is an enhancement in nematode locomotion, as quantified by thrashing rate. This cell-specific signaling pathway requires the transcription factor HSF-1, most likely through its transcriptional control of the α-crystallin ortholog, small heat shock protein HSP-16.48.

Our data support the interpretation that a G_αs_-signaling pathway is directly activated or modulated by ethanol. There is ample evidence in other systems for ethanol-induced activation of G-protein-coupled signaling pathways, at least *in vitro*. Ethanol can enhance GABA release and the extent of this enhancement can be regulated by various GPCRs, mostly via G_αi_-coupled receptors (Kelm *et al.* 2011). Ethanol activation of GABAergic release in the central amygdala, an area of the brain prominently associated with alcohol dependence, requires the G-protein-coupled CRH 1 receptor (Nie *et al.* 2004). Downstream of GPCRs, ethanol can directly activate specific forms of adenylyl cyclase (Yoshimura and Tabakoff 1995; Yoshimura *et al.* 2006), affect cellular cAMP levels (Rex *et al.* 2008; Gupta *et al.* 2013), activate PKA (Kelm *et al.* 2008; Wang *et al.* 2015), and induce protein phosphorylation (Conti *et al.* 2009). Genetic or pharmacological manipulation of individual components of this pathway has measurable effects on complex alcohol dependency phenotypes, as adenylyl cyclase mutants and knockouts affect ethanol sensitivities and consumption in both mice and *Drosophila* (Moore *et al.* 1998; Maas *et al.* 2005; Xu *et al.* 2012). Indeed, the role of cAMP in the ethanol-induced stimulation of locomotion of mice was recently reported using a selective phosphodiesterase-4 inhibitor (Balino *et al.* 2016). Despite evidence of the G_αs_-signaling pathway in other models, it has been relatively unexplored in *C. elegans*. In *C. elegans*, the CRH1 homolog *seb-3* has a characterized high-alcohol phenotype; however, *seb-3* is specifically expressed in head ganglia and nerve cords (Jee *et al.* 2013) and not sensory neurons such as the IL2 neurons. Our data use combinations of optogenetic, genetic, and pharmacological manipulation to trace the involvement of the entire G_αs_ -cAMP-PKA signaling pathway in the IL2 neurons in ethanol-dependent stimulation. Additionally, we extend this pathway to the downstream phosphorylation of the exocytotic protein UNC-18 and implicate HSP-16.48 as a novel regulatory protein.

Phosphorylation of synaptic proteins is a dynamic mechanism for the regulation of vesicle fusion and synaptic transmission. Many phosphorylation targets have been described for serine/threonine kinases, although mostly *in vitro*. Phosphorylation of Munc18 has been demonstrated *in vivo*, primarily by Protein Kinase C (de Vries *et al.* 2000; Craig *et al.* 2003). PKA has not previously been shown to phosphorylate Munc18 or UNC-18, despite strong evidence that it does phosphorylate other synaptic proteins such as cysteine string protein (Evans *et al.* 2001). Both protein kinase C and PKA phosphorylation consensus sequences have similar requirements of basic amino acids upstream of the target serine, and indeed the region upstream of Ser322 is rich in lysine residues (Edwards *et al.* 2012). Intriguingly, in rat pancreatic acini, PKC-dependent phosphorylation of Munc18C occurs in response to acute exposure to ethanol (Cosen-Binker *et al.* 2007). Physiologically, phosphorylation alters the kinetics of amperometric spikes, in particular reducing the quantal size of individual release events (Barclay *et al.* 2003). Munc18 phosphorylation contributes to short-term plasticity at the synapse by controlling post-tetanic potentiation (Genc *et al.* 2014) and UNC-18 phosphorylation contributes to thermosensory behaviors of *C. elegans* (Edwards *et al.* 2012). Biochemically, phosphorylation of this region of either UNC-18 or Munc18 reduces the binding affinity for closed-conformation syntaxin (Barclay *et al.* 2003; Edwards *et al.* 2012). However, the classically characterized closed-conformation mutation of Munc18, R39C, has no effect on the stimulatory alcohol phenotype (Johnson *et al.* 2013), arguing against the physiological effects of low ethanol concentrations being achieved through regulation of that particular interaction. The IL2 neurons release acetylcholine as a fast neurotransmitter (Lee *et al.* 2012) and connect into the main locomotor circuits via intermediary neurons (White *et al.* 1986). It is therefore possible that phosphorylation is altering synaptic vesicle fusion in the IL2 neurons, thereby shaping the patterns of electrical activity governing nematode motility.

Our data demonstrate that the α-crystallin ortholog HSP-16.48 acts directly downstream of HSF-1, indicating that the effects of HSF-1 in this phenotype are likely simply as a transcription factor driving constitutive HSP-16.48 expression. HSF-1 controls the expression of heat-inducible stress proteins such as the small HSPs, and HSP-16.48 specifically, as evidenced by numerous experiments (Hsu *et al.* 2003; Prahlad *et al.* 2008; Larance *et al.* 2011; Kourtis *et al.* 2012). Exposure to very high concentrations of ethanol can induce the expression of many heat shock proteins (Kwon *et al.* 2004; Pignataro *et al.* 2007; Urquhart *et al.* 2016) and overexpression of some HSPs can alter sensitivity to the sedative effects of ethanol (Awofala *et al.* 2011). In *C. elegans*, basal expression of *hsp-16.48* alters the sedative effects of high doses of ethanol, but its overexpression only partially rescues the *hsf-1(sy441)* mutant phenotype (Johnson *et al.* 2016). In contrast, our results indicate that *hsp-16.48* can completely restore the stimulatory phenotype for the *hsf-1(sy441)* mutant. Although there is little constitutive HSP-16.48 expressed, even in response to 400 mM ethanol, our RNAi experiments show that these small quantities are sufficient to affect whole-animal behavior. Small HSPs can be directly phosphorylated by PKA (Garrido *et al.* 2012), but it is currently unknown how HSP-16.48 regulates the G_αs_ -cAMP-PKA signaling pathway. It may be that HSP-16.48 is acting simply as a chaperone to preserve either structural conformation of individual proteins, protein–protein interactions, or possibly to assist in the phosphorylation event itself. Alternatively, HSP-16.48 may directly interact with specific components of the signaling pathway independent of its chaperone ability. Nonetheless, the correct functioning of HSP-16.48 in ethanol-dependent phenotypes explicitly required a seven amino acid region of the protein’s N-terminus, a region not found in other small HSPs linked to temperature stress tolerance (Kourtis *et al.* 2012), and it will be intriguing to determine the mechanistic role for this protein region.

Our experiments have used thrashing as a measure of locomotion (Gjorgjieva *et al.* 2014). The main rationale for this selection was the simplicity and reproducibility of the assay. It is unknown experimentally whether other measures of locomotion, such as body bends or locomotor speed, would be similarly affected; however, that speculation would be considered likely. High alcohol concentrations reduce all aspects of locomotion by similar extents including thrashing (Mitchell *et al.* 2007; Graham *et al.* 2009; Johnson *et al.* 2013), body bends (Johnson *et al.* 2016), and locomotor speed (Davies *et al.* 2003; Kapfhamer *et al.* 2008) and there is no reason to suspect differences with low alcohol concentrations. Exposure to high or low ethanol has no reported effect on reversals or ω-turns of *C. elegans* on agar (Mitchell *et al.* 2010), but high alcohol did increase spontaneous reversals in solution by ∼1 reversal per minute (Topper *et al.* 2014). While uncharacterized in this study, it is unlikely that a change in reversal frequency would be significant enough to influence rates as high as 110–120 thrashes per minute.

The internal concentration of ethanol in nematodes is reported to be ∼10% of external experimental levels due to poor penetration through the cuticle (Davies *et al.* 2003; Alaimo *et al.* 2012; Johnson *et al.* 2016). However, the structure of the IL2 neurons, with dendritic projections directly to the exterior of the body (White *et al.* 1986), would be in contact with the exact level of ethanol seen in solution. Therefore, it is unclear whether this low-alcohol phenotype represents a phenotypic response to 17 mM or a substantially reduced concentration. However, in either case this phenotype does indicate a neuronal cell signaling effect in *C. elegans* in response to ethanol at levels that would be seen in intoxicated humans. The stimulation of locomotion has been reproducibly demonstrated in *C. elegans* (Graham *et al.* 2009; Johnson *et al.* 2013) but is smaller than that seen in *Drosophila* (Wolf *et al.* 2002). It is difficult to determine whether there could be a further enhancement of locomotion with exposure to higher levels of ethanol as competing depressive and stimulatory effects of ethanol may begin to cancel each other out as concentration is increased. Indeed, there is no net effect of 100 mM external ethanol on locomotion rate (Davies *et al.* 2003; Mitchell *et al.* 2007; Graham *et al.* 2009; Johnson *et al.* 2016). It remains possible that the stimulatory effects of 17 mM ethanol are actually underrepresented here due to the antagonistic sedative effects of higher alcohol concentrations. Why would *C. elegans* increase their movement in response to 17 mM ethanol? The ecology of the species is not fully understood; however, some evidence points to *C. elegans* feeding on bacteria associated with decomposing material. However, unlike longer chain alcohols, evidence for ethanol acting as a chemotactic cue signaling a potential nutrient source is not well supported. Although we were unable to reproduce the results, some data point to ethanol acting as a low-level chemorepellent (Bargmann *et al.* 1993; Lee *et al.* 2009). It is therefore conceivable that the stimulus in locomotor rate is enhancing escape or avoidance of a potentially poisonous substance. Alternatively, the locomotor stimulus is merely reflective of an indirect activation of a neuronal G-protein signaling pathway that feeds into the circuitry governing nematode locomotion (Gjorgjieva *et al.* 2014).

Alcohol acts paradoxically as both a depressant and as a stimulant. These stimulatory effects in humans are thought to be more rewarding than sedative effects and thus may play a more prominent role in determining addiction. The differentiator model for risk to alcoholism is associated with a trade-off between stimulant and sedative effects (Hendler *et al.* 2013). We have shown a unique genetic link between the α-crystallin homolog HSP-16.48 and both the sedative (Johnson *et al.* 2016) and now also the stimulatory effects of alcohol, where an increase in HSP-16.48 expression biphasically both enhances ethanol-induced stimulation and decreases ethanol-induced sedation. Importantly, the expression of the α-crystallin chaperone is upregulated in mice strains with a high alcohol intake preference (Mulligan *et al.* 2006) as well as human alcohol addicts (Iwamoto *et al.* 2004), indicative that the phenotypic effects presented here may indeed reflect neuronal pathways and have biomedical relevance in higher organisms. This identification of an ethanol-dependent signaling pathway, from G-protein to the phosphorylation target of UNC-18, therefore presents novel targets for future pharmacological intervention that could be exploited to control the devastating physiological effects of alcohol.

## Supplementary Material

Supplemental material is available online at www.genetics.org/lookup/suppl/doi:10.1534/genetics.117.300119/-/DC1.
