# Supplementary figures and images for: Ethanol Stimulates Locomotion via a Gαs-Signaling Pathway in IL2 Neurons in Caenorhabditis elegans
Source: Genetics. 2017 Sep 25;207(3):1023–39. doi: 10.1534/genetics.117.300119 (PMC5676223; doi:10.1534/genetics.117.300119)

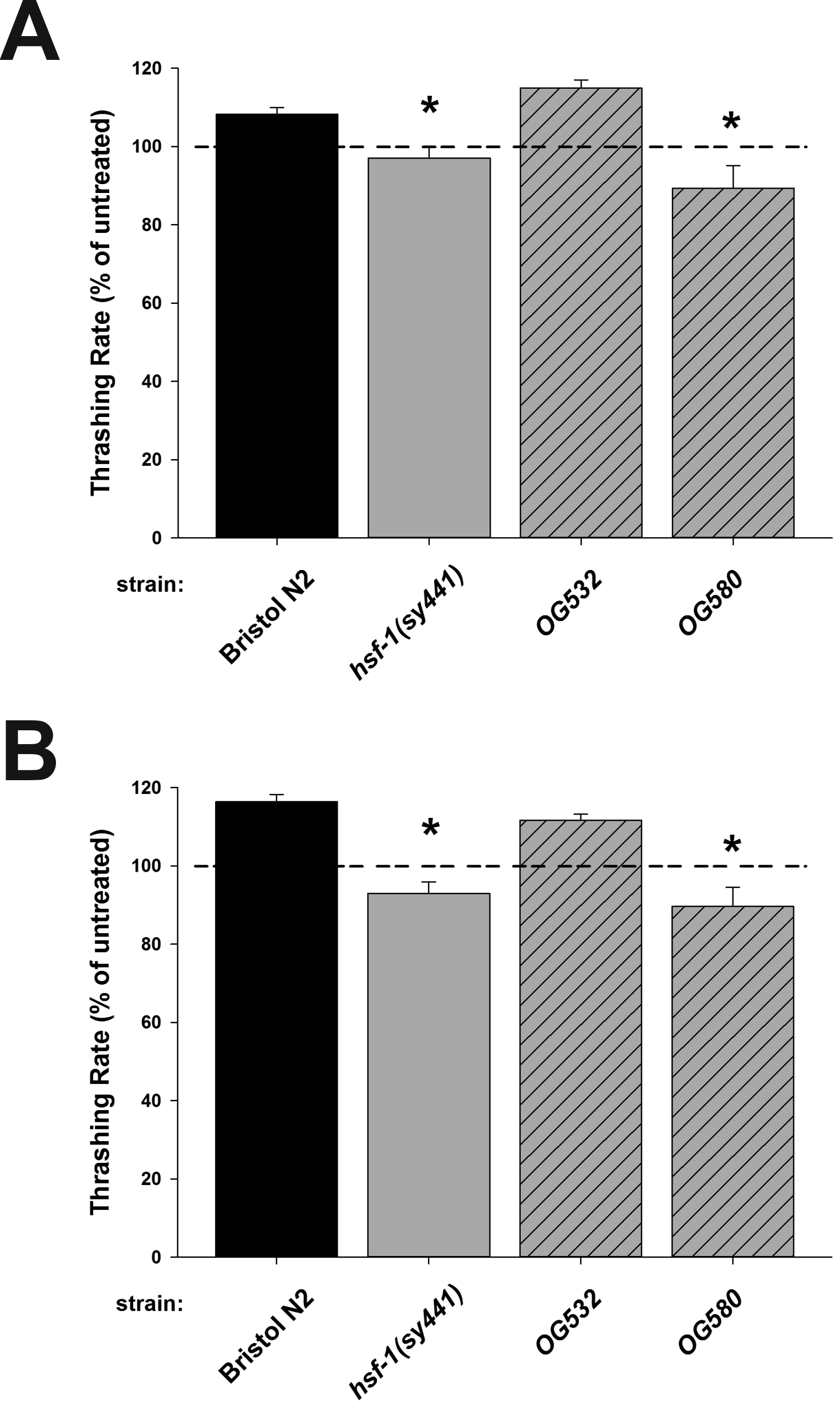

Supplement: Supplementary file 1 [file 1023FigureS1.tif]

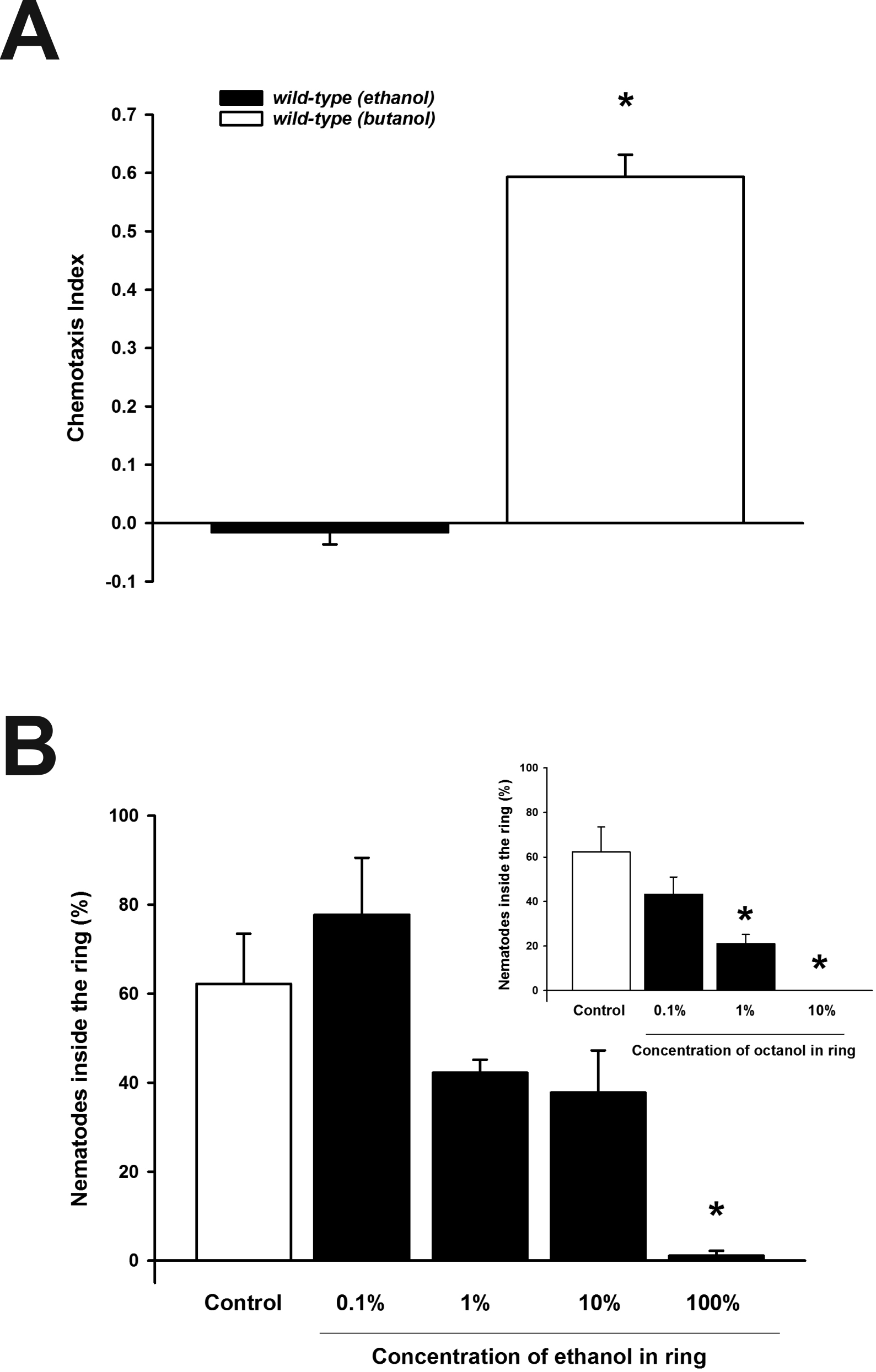

Supplement: Supplementary file 2 [file 1023FigureS2.tif]

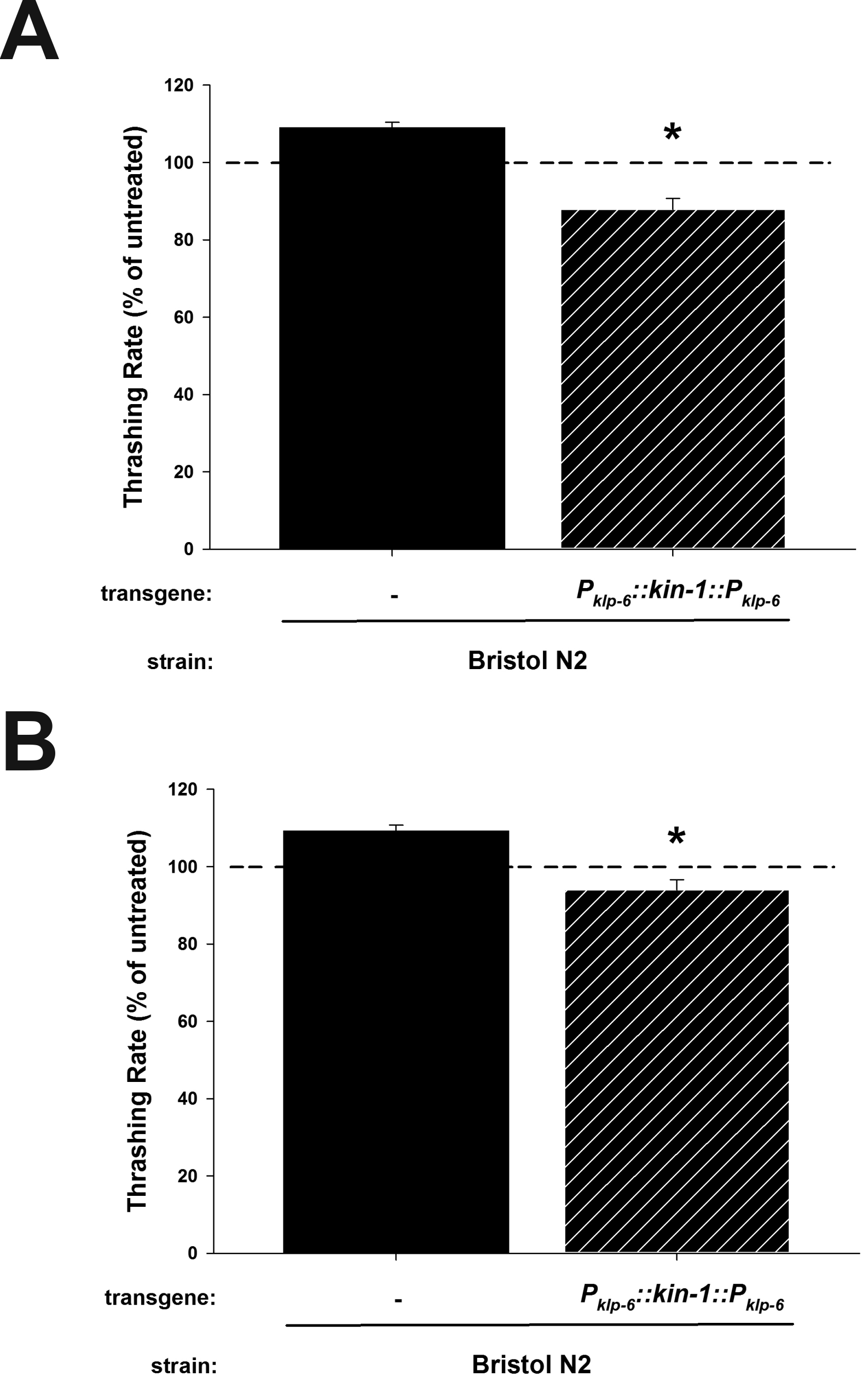

Supplement: Supplementary file 3 [file 1023FigureS3.tif]
