# Supplementary material for: Ethanol Stimulates Locomotion via a Gαs-Signaling Pathway in IL2 Neurons in Caenorhabditis elegans
Source: Genetics. 2017 Sep 25;207(3):1023–39. doi: 10.1534/genetics.117.300119 (PMC5676223; doi:10.1534/genetics.117.300119)
Supplement: Supplementary file 4 [file 1023FileS1.docx]

**TABLES**

**Table S1: Basal (untreated) thrashing rates of *C. elegans* strains used in this study.**

| *C. elegans* strain | **Locomotion Rate**  **(thrashes/min)** |
| --- | --- |
| Bristol N2 | 96.1±1.2 |
| (AMG160, 161, 162) Bristol N2;*Ex[P_hsf-1_::hsf-1]* | 83.8±2.0 |
| (AMG157, 158, 159) Bristol N2;*Ex[P_rab-3_::hsb-1]* | 83.3±2.9 |
| (AMG475, 476, 477) Bristol N2;*Ex[P_klp-6_::hsb-1]* | 97.9±2.4 |
| (AMG109, 110, 111) Bristol N2;*Ex[P_rab-3_::hsp-16.48]* | 75.5±4.2 |
| (AMG142, 143, 144) Bristol N2;*Ex[P_rab-3_::hsp-16.48Δ38-44]* | 68.4±4.9 |
| (AMG55, 56, 57) Bristol N2;*Ex[P_unc-18_::unc-18 S322A]* | 83.2±2.5 |
| (AMG478, 479, 480) Bristol N2;*Ex[P_klp-6_::unc-18 S322A]* | 101.3±1.9 |
| (AMG481, 482, 483) Bristol N2;*Ex[P_klp-6_::kin-1::P_klp-6_]* | 89.7±2.8 |
| PS3551 *hsf-1(sy441)* | 70.1±1.6 |
| (AMG73, 74, 75) *hsf-1(sy441);Ex[P_hsf-1_::hsf-1]* | 74.4±2.1 |
| (AMG76, 77, 78) *hsf-1(sy441);Ex[P_rab-3_::hsf-1]* | 66.6±3.3 |
| (AMG79, 80, 81) *hsf-1(sy441);Ex[P_myo-3_::hsf-1]* | 61.5±3.4 |
| (AMG85, 86, 87) *hsf-1(sy441);Ex[P_glr-1_::hsf-1]* | 67.8±2.3 |
| (AMG82, 83, 84) *hsf-1(sy441);Ex[P_unc-17_::hsf-1]* | 65.3±2.6 |
| (AMG91, 92, 93) *hsf-1(sy441);Ex[P_gcy-8_::hsf-1]* | 61.6±2.2 |
| (AMG88, 89, 90) *hsf-1(sy441);Ex[P_osm-6_::hsf-1]* | 60.9±4.1 |
| (AMG94, 95, 96) *hsf-1(sy441);Ex[P_klp-6_::hsf-1]* | 80.8±4.3 |
| (AMG97, 98, 99) *hsf-1(sy441);Ex[P_rab-3_::hsp-16.48]* | 64.7±2.0 |
| (AMG469, 470, 471) *hsf-1(sy441);Ex[P_rab-3_::hsp-16.48 Δ38-44]* | 63.2±2.8 |
| (AMG472, 473, 474) *hsf-1(sy441);Ex[P_klp-6_::hsp-16.48]* | 70.2±2.7 |
| OG532 *hsf-1(sy441);drSi13[hsf-1p::hsf-1::GFP::unc-54 3’UTR + Cbr-unc-119(+)]* | 87.7±6.8 |
| OG580 *hsf-1(sy441);drSi28[hsf-1p::hsf-1(R145A)::GFP::unc-54 3’UTR + Cbr-unc-119(+)]* | 53.6±4.1 |
| MT363 *goa-1(n363)* | 63.1±4.0 |
| NM1380 *egl-30(js126)* | 70.0±2.5 |
| KG524 *gsa-1(ce94)* | 133.3±7.9 |
| KG421 *gsa-1(ce81)* | 152.9±5.4 |
| KG1180 *lite-1(ce314)* | 47.2±0.9 |
| AMG484 *lite-1(ce314);Ex[Pklp-6::JellyOp]* | 40.7±0.9 |
| AMG 487 *lite-1(ce314);Ex[Pklp-6::hRh1]* | 38.2±1.1 |
| NL2099 *rrf-3(pk1426) (empty vector RNAi)* | 92.1±2.5 |
| NL2099 *rrf-3(pk1426) (hsf-1 RNAi)* | 93.7±1.8 |
| NL2099 *rrf-3(pk1426) (hsp-16.48 RNAi)* | 88.7±2.5 |

**Table S2: Thrashing rates of individual transgenic lines used in this study.**

| *C. elegans* transgenic line | **Locomotion Rate – untreated (thrashes per minute)** | **Locomotion Rate – ethanol (thrashes per minute)** |
| --- | --- | --- |
| AMG160 Bristol N2;*ulvEx160[P_hsf-1_::hsf-1]* | 76.0±2.4 | 91.2±4.7 |
| AMG161 Bristol N2;*ulvEx161[P_hsf-1_::hsf-1]* | 87.5±3.6 | 95.1±3.9 |
| AMG162 Bristol N2;*ulvEx162[P_hsf-1_::hsf-1]* | 87.9±3.5 | 94.3±3.6 |
| AMG157 Bristol N2;*ulvEx157[P_rab-3_::hsb-1]* | 85.5±3.1 | 76.7±5.3 |
| AMG158 Bristol N2;*ulvEx158[P_rab-3_::hsb-1]* | 73.4±5.8 | 64.4±7.9 |
| AMG159 Bristol N2;*ulvEx159[P_rab-3_::hsb-1]* | 92.0±3.5 | 84.9±4.5 |
| AMG475 Bristol N2;*ulvEx475[P_klp-6_::hsb-1]* | 96.4±5.4 | 92.0±4.0 |
| AMG476 Bristol N2;*ulvEx476[P_klp-6_::hsb-1]* | 97.5±4.3 | 95.4±4.3 |
| AMG477 Bristol N2;*ulvEx477[P_klp-6_::hsb-1]* | 99.9±2.8 | 85.5±4.9 |
| AMG109 Bristol N2;*ulvEx109[P_rab-3_::hsp-16.48]* | 74.8±6.8 | 103.8±4.4 |
| AMG110 Bristol N2;*ulvEx110[P_rab-3_::hsp-16.48]* | 76.6±4.8 | 90.9±4.0 |
| AMG111 Bristol N2;*ulvEx111[P_rab-3_::hsp-16.48]* | 76.1±7.5 | 82.9±5.1 |
| AMG142 Bristol N2;*ulvEx142[P_rab-3_::hsp-16.48Δ38-44]* | 55.8±6.3 | 47.6±3.8 |
| AMG143 Bristol N2;*ulvEx143[P_rab-3_::hsp-16.48Δ38-44]* | 80.9±5.1 | 77.5±5.5 |
| AMG144 Bristol N2;*ulvEx144[P_rab-3_::hsp-16.48Δ38-44]* | 71.0±3.2 | 63.4±10.2 |
| AMG55 Bristol N2;*ulvEx55[P_unc-18_::unc-18 S322A]* | 84.8±4.4 | 79.4±3.6 |
| AMG56 Bristol N2;*ulvEx56[P_unc-18_::unc-18 S322A]* | 76.2±3.0 | 72.2±3.5 |
| AMG57 Bristol N2;*ulvEx57[P_unc-18_::unc-18 S322A]* | 88.6±4.8 | 83.6±2.5 |
| AMG478 Bristol N2;*ulvEx478[P_klp-6_::unc-18 S322A]* | 104.2±3.7 | 101.5±4.7 |
| AMG479 Bristol N2;*ulvEx479[P_klp-6_::unc-18 S322A]* | 96.4±2.8 | 93.5±5.0 |
| AMG480 Bristol N2;*ulvEx480[P_klp-6_::unc-18 S322A]* | 103.4±2.7 | 98.3±3.0 |
| AMG481 Bristol N2;*ulvEx481[P_klp-6_::kin-1::P_klp-6_]* | 84.2±2.9 | 75.0±3.6 |
| AMG482 Bristol N2;*ulvEx482[P_klp-6_::kin-1::P_klp-6_]* | 103.3±4.8 | 89.8±4.7 |
| AMG483 Bristol N2;*ulvEx483[P_klp-6_::kin-1::P_klp-6_]* | 81.7±3.8 | 71.7±5.0 |
| AMG73 *hsf-1(sy441);ulvEx73[P_hsf-1_::hsf-1]* | 64.6±4.3 | 66.4±3.5 |
| AMG74 *hsf-1(sy441);ulvEx74[P_hsf-1_::hsf-1]* | 84.8±3.1 | 90.2±2.8 |
| AMG75 *hsf-1(sy441);ulvEx75[P_hsf-1_::hsf-1]* | 66.0±3.2 | 82.0±2.8 |
| AMG76 *hsf-1(sy441);ulvEx76[P_rab-3_::hsf-1]* | 79.8±3.6 | 91.4±2.1 |
| AMG77 *hsf-1(sy441);ulvEx77[P_rab-3_::hsf-1]* | 80.2±3.7 | 90.2±3.9 |
| AMG78 *hsf-1(sy441);ulvEx78[P_rab-3_::hsf-1]* | 64.8±5.1 | 108.4±2.4 |
| AMG79 *hsf-1(sy441);ulvEx79[P_myo-3_::hsf-1]* | 58.9±6.1 | 55.6±8.6 |
| AMG80 *hsf-1(sy441);ulvEx80[P_myo-3_::hsf-1]* | 66.2±8.3 | 58.2±6.0 |
| AMG81 *hsf-1(sy441);ulvEx81[P_myo-3_::hsf-1]* | 59.4±2.4 | 52.6±2.3 |
| AMG85 *hsf-1(sy441);ulvEx85[P_glr-1_::hsf-1]* | 71.3±6.3 | 66.9±5.1 |
| AMG86 *hsf-1(sy441);ulvEx86[P_glr-1_::hsf-1]* | 67.4±2.2 | 60.0±2.1 |
| AMG87 *hsf-1(sy441);ulvEx87[P_glr-1_::hsf-1]* | 64.6±2.2 | 58.6±2.5 |
| AMG82 *hsf-1(sy441);ulvEx82[P_unc-17_::hsf-1]* | 69.3±5.9 | 94.1±5.4 |
| AMG83 *hsf-1(sy441);ulvEx83[P_unc-17_::hsf-1]* | 62.4±4.6 | 75.6±1.9 |
| AMG84 *hsf-1(sy441);ulvEx84[P_unc-17_::hsf-1]* | 64.2±2.5 | 69.4±1.7 |
| AMG91 *hsf-1(sy441);ulvEx91[P_gcy-8_::hsf-1]* | 65.8±5.6 | 58.8±6.8 |
| AMG92 *hsf-1(sy441);ulvEx92[P_gcy-8_::hsf-1]* | 58.0±2.1 | 49.8±1.8 |
| AMG93 *hsf-1(sy441);ulvEx93[P_gcy-8_::hsf-1]* | 61.0±2.7 | 50.2±2.0 |
| AMG88 *hsf-1(sy441);ulvEx88[P_osm-6_::hsf-1]* | 62.5±7.8 | 69.9±4.3 |
| AMG89 *hsf-1(sy441);ulvEx89[P_osm-6_::hsf-1]* | 63.1±8.0 | 75.6±4.4 |
| AMG90 *hsf-1(sy441);ulvEx90[P_osm-6_::hsf-1]* | 57.0±5.9 | 68.0±3.0 |
| AMG94 *hsf-1(sy441);ulvEx94[P_klp-6_::hsf-1]* | 79.7±4.0 | 92.5±4.1 |
| AMG95 *hsf-1(sy441);ulvEx95[P_klp-6_::hsf-1]* | 80.9±4.3 | 89.4±3.1 |
| AMG96 *hsf-1(sy441);ulvEx96[P_klp-6_::hsf-1]* | 80.8±5.7 | 96.4±5.3 |
| AMG97 *hsf-1(sy441);ulvEx97[P_rab-3_::hsp-16.48]* | 63.5±3.9 | 73.1±4.5 |
| AMG98 *hsf-1(sy441);ulvEx98[P_rab-3_::hsp-16.48]* | 65.2±2.9 | 78.5±4.1 |
| AMG99 *hsf-1(sy441);ulvEx99[P_rab-3_::hsp-16.48]* | 65.6±3.6 | 77.1±3.3 |
| AMG469 *hsf-1(sy441);ulvEx469[P_rab-3_::hsp-16.48 Δ38-44]* | 58.6±3.7 | 50.9±6.3 |
| AMG470 *hsf-1(sy441);ulvEx470[P_rab-3_::hsp-16.48 Δ38-44]* | 62.0±4.7 | 60.5±6.9 |
| AMG471 *hsf-1(sy441);ulvEx471[P_rab-3_::hsp-16.48 Δ38-44]* | 68.9±5.7 | 58.5±4.9 |
| AMG472 *hsf-1(sy441);ulvEx472[P_klp-6_::hsp-16.48]* | 67.2±4.5 | 73.5±3.5 |
| AMG473 *hsf-1(sy441);ulvEx473[P_klp-6_::hsp-16.48]* | 76.4±5.2 | 88.2±3.5 |
| AMG474 *hsf-1(sy441);ulvEx474[P_klp-6_::hsp-16.48]* | 66.9±4.3 | 84.2±4.3 |
