# Supplementary material for: Ethanol Stimulates Locomotion via a Gαs-Signaling Pathway in IL2 Neurons in Caenorhabditis elegans
Source: Genetics. 2017 Sep 25;207(3):1023–39. doi: 10.1534/genetics.117.300119 (PMC5676223; doi:10.1534/genetics.117.300119)
Supplement: Supplementary file 5 [file 1023FileS2.docx]

**Johnson *et al*. Ethanol stimulates locomotion via a G_αs_-signalling pathway in IL2 neurons in *C. elegans***

**Supplemental Figure Legends**

**Figure S1.** Single-copy rescue of *hsf-1(sy441)* rescues the lack of ethanol and forskolin stimulation of locomotion. **(A)** The absence of ethanol stimulation in *hsf-1(sy441)* mutants was restored by single-copy expression of wild-type *hsf-1* (OG532), but not by single-copy expression of a DNA binding defective mutant of *hsf-1* (OG580). OG532 and OG580 strains were generated by Morton and Lamitina (2013). **(B)** The absence of forskolin stimulation in *hsf-1(sy441)* mutants was restored by single-copy expression of wild-type *hsf-1* (OG532), but not by single-copy expression of a DNA binding defective mutant of *hsf-1* (OG580). In both (A) and (B), data are expressed normalised to untreated controls. For each experiment, exposure to ethanol or forskolin enhanced the locomotion rate of Bristol N2 worms (Mann-Whitney U-test; P<0.05). * indicates significant difference in comparison to untreated Bristol N2. Comparisons were made by one-way analysis of variance with Tukey post-hoc comparisons (P<0.001; N = 30 for each condition). Bristol N2 worms are depicted in black, *hsf-1(sy441)* in grey. Hatching indicates single-copy rescue of *hsf-1(sy441*).

**Figure S2.** Ethanol does not act as a chemoattractant or chemorepellant. **(A)** In a chemotaxis assay, exposure to 100% ethanol had no effect on movement of Bristol N2 worms within 90 minutes. In comparison, 100% butanol acted as a strong chemoattractant. Comparison was made by t-test. *; P < 0.001 (t-test); N = 3 (butanol) and 6 (ethanol). **(B)** In an avoidance assay, Bristol N2 worms only avoided ethanol at concentrations greater than 10%. In contrast, worms avoided octanol at concentrations as low as 1% (***Inset***). * indicates significant difference in comparison to control conditions. Comparisons were made by one-way analysis of variance with Tukey post-hoc comparisons (P<0.001; N = 3). Control worms (exposed to distilled water) are depicted in white, alcohol exposed worms in black.

**Figure S3.** IL2 neuron-specific RNAi of protein kinase A (PKA) blocks ethanol and forskolin stimulation of locomotion. **(A)** IL2 neuron-specific knockdown of the catalytic subunit of *C. elegans* PKA (*kin-1*) blocked the ethanol phenotype in Bristol N2 worms. **(B)** IL2 neuron-specific knockdown of the catalytic subunit of *C. elegans* PKA (*kin-1*) blocked the forskolin phenotype in Bristol N2 worms. In both (A) and (B), data are expressed normalised to untreated controls. For each experiment, exposure to ethanol or forskolin enhanced the locomotion rate of Bristol N2 worms (Mann-Whitney U-test; P<0.05). * indicates significant difference in comparison to untreated Bristol N2. Comparisons were made by one-way analysis of variance with Tukey post-hoc comparisons (P<0.001; N = 30 for each condition). Bristol N2 worms are depicted in black. Hatching indicates *P_klp-6_::kin-1::P_klp-6_* expression.

**Table S1.** Basal (untreated) thrashing rates of *C. elegans* strains used in this study. For strains grouped in parentheses, please see Table S2 for the genotypes of the individual independent transgenic lines.

**Table S2**. Thrashing rates of individual transgenic lines used in this study.
